# Co-opting templated aggregation to degrade pathogenic tau assemblies and improve motor function

**DOI:** 10.1016/j.cell.2024.08.024

**Published:** 2024-09-13

**Authors:** Lauren V.C. Miller, Guido Papa, Marina Vaysburd, Shi Cheng, Paul W. Sweeney, Annabel Smith, Catarina Franco, Taxiarchis Katsinelos, Melissa Huang, Sophie A.I. Sanford, Jonathan Benn, Jasmine Farnsworth, Katie Higginson, Holly Joyner, William A. McEwan, Leo C. James

**Affiliations:** 1 UK Dementia Research Institute at the University of Cambridge, Department of Clinical Neurosciences, Hills Road, Cambridge CB2 0AH, UK; 2 MRC Laboratory of Molecular Biology, Francis Crick Avenue, Cambridge CB2 0QH, UK; 3 Cancer Research UK Cambridge Institute, Robinson Way, Cambridge CB2 0RE, UK; 4 GMU-GIBH Joint School of Life Sciences, The Guangdong-Hong Kong-Macau Joint Laboratory for Cell Fate, Regulation and Diseases, Guangzhou Medical University, Guangzhou, China

## Abstract

Protein aggregation causes a wide range of neurodegenerative diseases. Targeting and removing aggregates, but not the functional protein, is a considerable therapeutic challenge. Here, we describe a therapeutic strategy called “RING-Bait,” which employs an aggregating protein sequence combined with an E3 ubiquitin ligase. RING-Bait is recruited into aggregates, whereupon clustering dimerizes the RING domain and activates its E3 function, resulting in the degradation of the aggregate complex. We exemplify this concept by demonstrating the specific degradation of tau aggregates while sparing soluble tau. Unlike immunotherapy, RING-Bait is effective against both seeded and cell-autonomous aggregation. RING-Bait removed tau aggregates seeded from Alzheimer’s disease (AD) and progressive supranuclear palsy (PSP) brain extracts and was also effective in primary neurons. We used a brain-penetrant adeno-associated virus (AAV) to treat P301S tau transgenic mice, reducing tau pathology and improving motor function. A RING-Bait strategy could be applied to other neurodegenerative proteinopathies by replacing the Bait sequence to match the target aggregate.

## Graphical abstract

**Graphical abstract:**
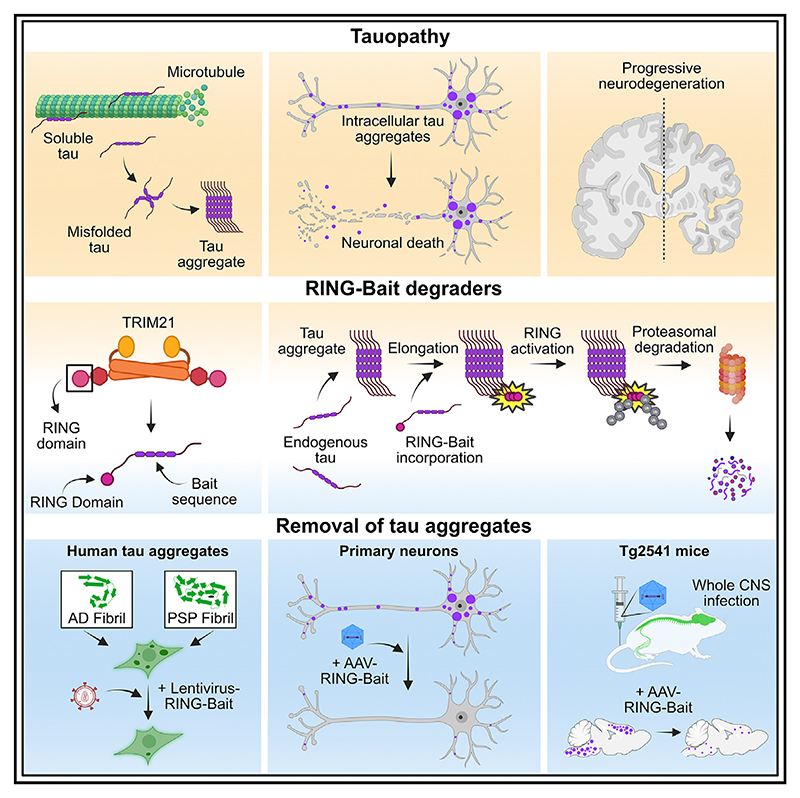

## Introduction

The formation of proteins into ordered fibrillar aggregates is the predominant molecular pathology in most neurodegenerative diseases. The microtubule-associated protein tau forms cytosolic assemblies in a number of diseases, including Alzheimer’s disease (AD) and progressive supranuclear palsy (PSP). Cryoelectron microscopy has recently revealed that tau fibrils adopt distinct conformations in particular diseases, potentially linking these conformations with disease-specific pathophysiology.^1^ Rare mutations in the *MAPT* gene that encodes tau can cause dominantly inherited frontotemporal dementias with tau pathology.^2^ This provides strong evidence that the aggregation of tau can drive disease progression in rare tauopathies and potentially also in more common sporadic tauopathies. Other proteins implicated in neurodegenerative diseases, such as TDP-43 and a-synuclein, can also adopt cytosolic fibrillar aggregates with disease-specific conformations.^3,4^ Based on this evidence, considerable effort is being expended to prevent or reverse ordered protein aggregation as a disease-modifying therapeutic strategy for neurodegenerative diseases.

The cytosolic aggregates that arise from the pathological formation of tau range from small, soluble, seed-competent species to large, insoluble filaments. Their effective removal therefore represents a difficult problem for the cell. Therapeutic intervention targeting tau has been met without clinical success.^5^ The most common drugs in clinical trials are monoclonal antibodies, which are generally delivered to the periphery and are required to access the CNS for target engagement. This presents a challenge given that the blood-brain barrier excludes most antibodies from the brain parenchyma and that the major site of protein aggregation, the cytosol, is very poorly accessed by antibodies.^6^ For tau, strategies have focused on binding extracellular protein aggregates to inhibit seeded aggregation or the prion-like spread of tau pathology, which is proposed to contribute to pathological progression. This approach, however, does not lend itself to removing existing intraneuronal pathology, and it is still unclear to what extent seeded aggregation drives disease in different tauopathies.^7–9^ Other approaches such as proteolyis-targeting chimeras (PROTACs), which recruit ubiquitination machinery to aggregates, have shown potential in degrading tau in cell-based models; however, they also suffer from poor bioavailability and aggregate specificity, as well as complex pharmacodynamics.^10,11^

We have previously described TRIM21 as a cytoplasmic antibody receptor and E3 ubiquitin ligase.^12^ Structures such as virus particles and tau assemblies that enter the cytosol with antibodies attached are rapidly bound by TRIM21.^12,13^ The stoichiometric clustering of TRIM21 following cytosolic immune complex engagement activates the ubiquitin E3 ligase activity of the TRIM21 RING domain, resulting in the recruitment of cellular degradation machinery such as the AAA ATPase VCP/p97 and the proteasome.^14,15^ In the case of tau, this reduces seeded aggregation in cells and ameliorates pathology in a mouse model of tauopathy.^13,16^ However, while this process can target incoming tau seeds, antibodies do not permit the degradation of pre-existing intracellular aggregates. Developing intracellular degraders would overcome this problem and allow the destruction of both pre-existing pathology and the ongoing pathological accumulation of tau assemblies. A key challenge facing intracellular degraders against neurodegenerative aggregates is achieving specificity for the aggregated species over the soluble monomers so that only the misfolded disease-causing form of a protein is removed and not the functional protein pool. We therefore sought to develop an intracellular, aggregate-specific degradation system.

We reasoned that homotypic protein aggregation represents an opportunity to selectively incorporate effector domains to redirect fibrils for degradation. By fusing the RING domain of TRIM21 to a tau “Bait,” the catalytic activity of TRIM21 is brought directly to fibrils without the requirement of intracellular antibodies. TRIM21 has established activity as an E3 ligase against complex cytosolic substrates including virus particles and seed-competent tau assemblies. We therefore hypothesized that recruitment of the TRIM21 RING domain to growing tau fibrils, a process that occurs both during cell-autonomous and seeded aggregation, would lead to the degradation of these assemblies. Importantly, owing to the clustering requirement for activation, monomeric tau should be spared. Here, we show that this approach successfully removes ~90% of oligomerized tau species in HEK model systems and more than 60% in mouse brains after delivery of a RING-Bait construct using an adeno-associated viral vector. This technology paves the way for the development of gene therapy-based treatments for neurodegenerative diseases characterized by the accumulation of intracellular protein aggregates and can be used as a screening tool to test whether protein aggregates are amenable to proteasomal degradation without the requirement for target-specific binding domains, such as nanobodies.

## Results

### RING-Bait technology efficiently removes tau aggregates while sparing soluble endogenous proteins

**Figure 1:**
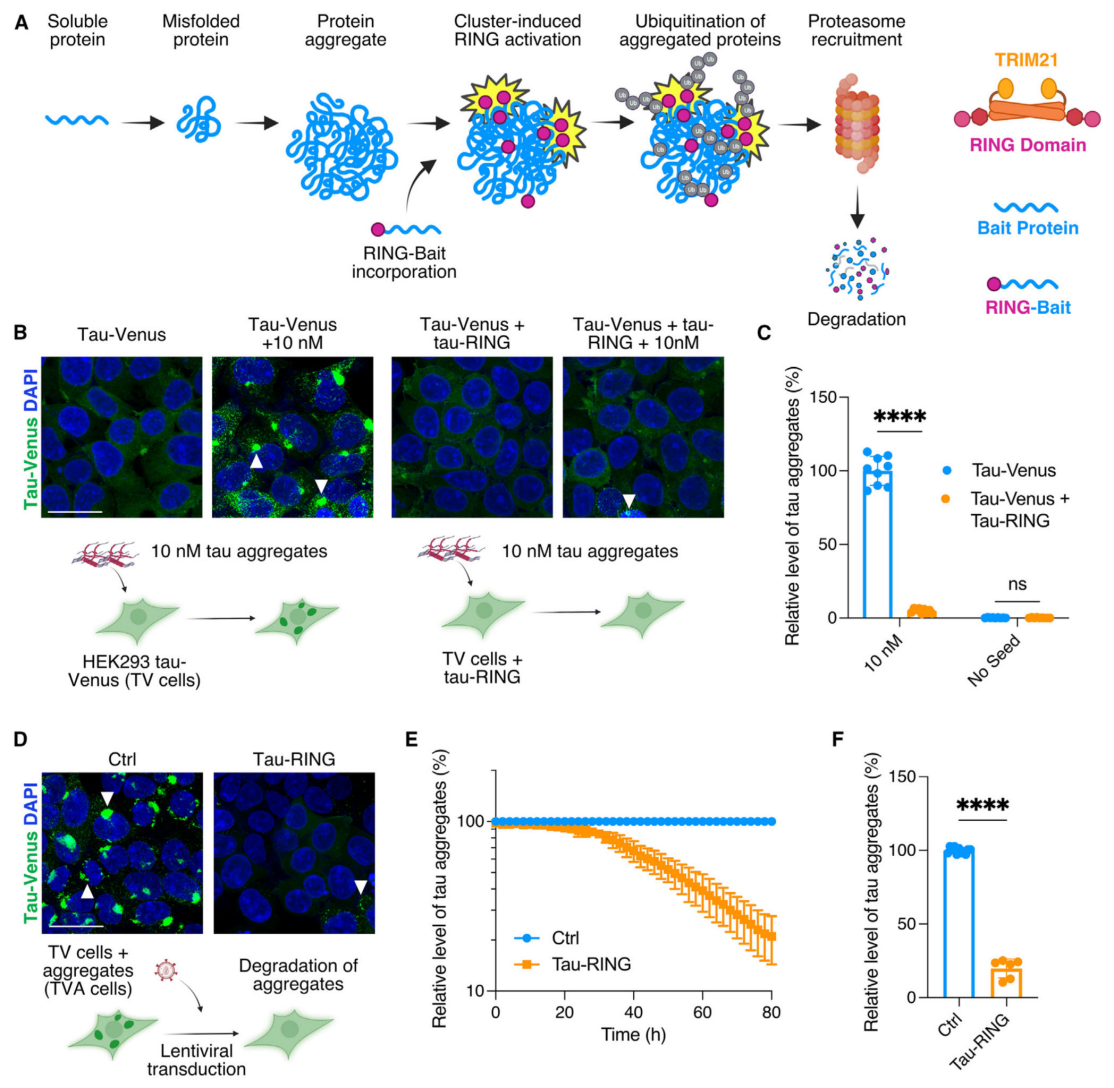
Tau-RING prevents seeded aggregation and removes existing aggregates (A) Schematic of RING-Bait technology. (B) Representative confocal images of HEK293 reporter cell line expressing tau-venus (TV cells) ± tau-RING, seeded ±10 nM tau aggregates with Lipofectamine 2000. White arrows denote examples of aggregates. (C) Quantification of live-cell images from cells treated as in (B). *n* = 3. (D) Representative confocal images of HEK293 reporter cell line expressing tau-venus, constitutively bearing tau aggregates (TVA cells), infected with lentivirus containing tau-RING and evaluated after 72 h. (E) Time course of tau aggregates in TVA reporter cells with and without tau-RING. *n* = 3. (F) Quantification of the number of aggregates in TVA cells 72 h post transduction with tau-RING lentivirus from live-cell images. *N* = 3. Scale bars, 25 μm. Statistical significance for (C) determined by two-way ANOVA and Sidak’s multiple comparison test. Statistical significance for (F) determined by unpaired t test. ****p < 0.0001. ns, non-significant. See also Figures S1 and S2.

The RING-Bait technology relies on the intracellular expression of a fusion protein composed of a Bait domain, which is identified as the protein that aggregates, directly fused to the RING domain of the E3 ligase TRIM21 (Figure 1A). To exemplify RING-Bait technology, we selected tau as both the target of degradation and the Bait. We generated a chimeric protein containing the TRIM21 RING domain fused to the C-terminal end of full-length 0N4R tau with the P301S mutation. The P301S mutation causes early-onset familial dementia and is pro-aggregant, increasing the rate of tau fibril extension by approximately 50-fold compared with wild-type (WT) tau.^17^ The C-terminus was chosen for the location of the RING domain owing to existing data indicating P301S tau aggregation is not significantly affected by C-terminal fusions.^13^ We reasoned that during the process of fibril elongation tau-RING will be incorporated into growing tau aggregates, activating TRIM21-RING-dependent ubiquitination, leading to subsequent degradation of the aggregate.

In order to test our hypothesis, we used HEK293 cells stably expressing P301S 0N4R tau fused to venus fluorescent protein (hereafter referred to as “TV cells”). TV cells have previously been shown to respond to exogenously supplied tau aggregates in the presence of transfection reagents by the formation of large, bright tau-venus puncta, which are sarkosyl insoluble (SI) upon extraction.^13^ These large tau-venus positive puncta are composed of multiple fibrillar tau aggregates when resolved by higher resolution microscopy^18^ (Figure S1A). The puncta can be segmented and quantified using live-cell imaging, enabling the creation of a high-throughput assay that can monitor aggregate levels longitudinally (Figure S1B).

TV cells were transduced with lentivirus to stably express tau-RING protein. We tested whether expression of tau-RING inhibited the seeded aggregation of tau-venus following transfection of exogenous tau assemblies. Recombinant 0N4R P301S tau was aggregated with heparin and supplied to the cells in the presence of Lipofectamine 2000. Tau-venus aggregates were quantified after 72 h. The tau-RING construct reduced seeded aggregation in TV cells by <95% compared with cells that did not express tau-RING (Figures 1B, 1C, and S1C).

To investigate if tau-RING is acting specifically on seeded tau aggregates or is simply reducing the available pool of intracellular tau, we analyzed the total levels of tau-venus upon co-expression with tau-RING (Figure S1E). No significant effect on soluble tau-venus was observed in the presence of tau-RING, suggesting the construct was not degrading monomeric tau (Figure S1F). Upon seeding, no significant difference in tau-venus levels was observed. By contrast, a significant reduction in the levels of tau phosphorylated at the S202 and T205 sites was seen in cells expressing tau-RING, as detected by the antibody AT8 (Figures S1G–S1J). In order to demonstrate that tau-RING was capable of aggregation and thus unlikely to be preventing seeding by steric inhibition, we expressed and purified 0N4R tau and 0N4R tau-RING protein, both with the P301S mutation. Using transmission electron microscopy (TEM), we confirmed that tau-RING and tau both formed bona fide fibrillar aggregates in the presence of heparin. Furthermore, the aggregation kinetics of tau-RING in the presence of heparin was comparable to unmodified P301S tau (Figures S1K and S1L).

Having demonstrated that tau-RING can reduce seeded tau aggregation, we investigated whether it could also be deployed in cells with pre-existing aggregates. To this end, we used TV cells that had been previously seeded with tau assemblies and constitutively propagate P301S tau-venus in an aggregated state (hereafter named “TVA” cells). These cells were then transduced with a lentivirus encoding tau-RING and monitored for the presence of tau-venus puncta over time (Figure 1D). We observed an >80% reduction in tau-venus positive aggregates over 72 h (Figures 1E, 1F, and S1D). By contrast, control lentiviruses expressing tau-mCherry or a non-specific nanobody-RING construct did not affect the number of tau-venus aggregates (Figures S1M and S1N). We hypothesize that as the elongation of tau fibrils occurs during both seeded aggregation and during the replication of pre-existing assemblies, this shared mechanism of incorporation of tau-RING is what drives the successful removal of new and pre-existing intracellular fibrils.

The results thus far are consistent with an underlying mechanism of tau-RING being incorporated into growing tau assemblies prior to mediating their destruction. To directly observe this, we engineered baby hamster kidney (BHK) cells to express P301S tau-venus and a doxycycline-inducible P301S tau-RING fused to mCherry fluorescent protein (Figure S2A). This cell line was chosen owing to its large cytoplasm, allowing real-time monitoring of the fate of cytosolic aggregates. We seeded these cells with heparin-assembled P301S tau seeds and allowed tauvenus aggregates to establish for 24 h prior to the addition of doxycycline. Tau-mCherry-RING was recruited to the site of existing tau-venus aggregates, consistent with its incorporation into growing fibrils (Figures S2B and S2C). Upon co-localization in an aggregate, tau-venus and tau-mCherry-RING were removed with similar kinetics (Figure S2D).

To determine if the removal of visible tau-venus aggregates resulted in the complete disassembly of the aggregate and did not produce smaller seed-competent species that could go on to further propagate, a “secondary seeding” assay was performed. In this assay, the lysate from TVA cells, infected with or without tau-mCherry-RING coding lentivirus, was probed for seed-competent species by adding the lysate onto fresh TV cells (Figure S2E). TVA lysate seeded abundant aggregation in fresh TV cells (Figures S2F and S2G). TVA + tau-mCherry-RING lysate seeded ~90% less aggregation than TVA lysate, demonstrating that the reduction in tau-venus aggregates, which could be observed visually closely, correlated with the number of seed-competent species present within cells (Figure S2G). TV lysate seeded no aggregation, confirming tau-venus does not aggregate to a detectable level independently in TV cells (Figure S2G). Taken together, these data suggest that tau-RING is recruited into growing aggregates, enabling it to target both seeded aggregation and pre-existing aggregates. Removal of an aggregate after incorporation of tau-RING results in an overall reduction in the number of seed-competent species that can continue to propagate.

### RING-Bait removes aggregates by initiating proteasomal degradation

**Figure 2:**
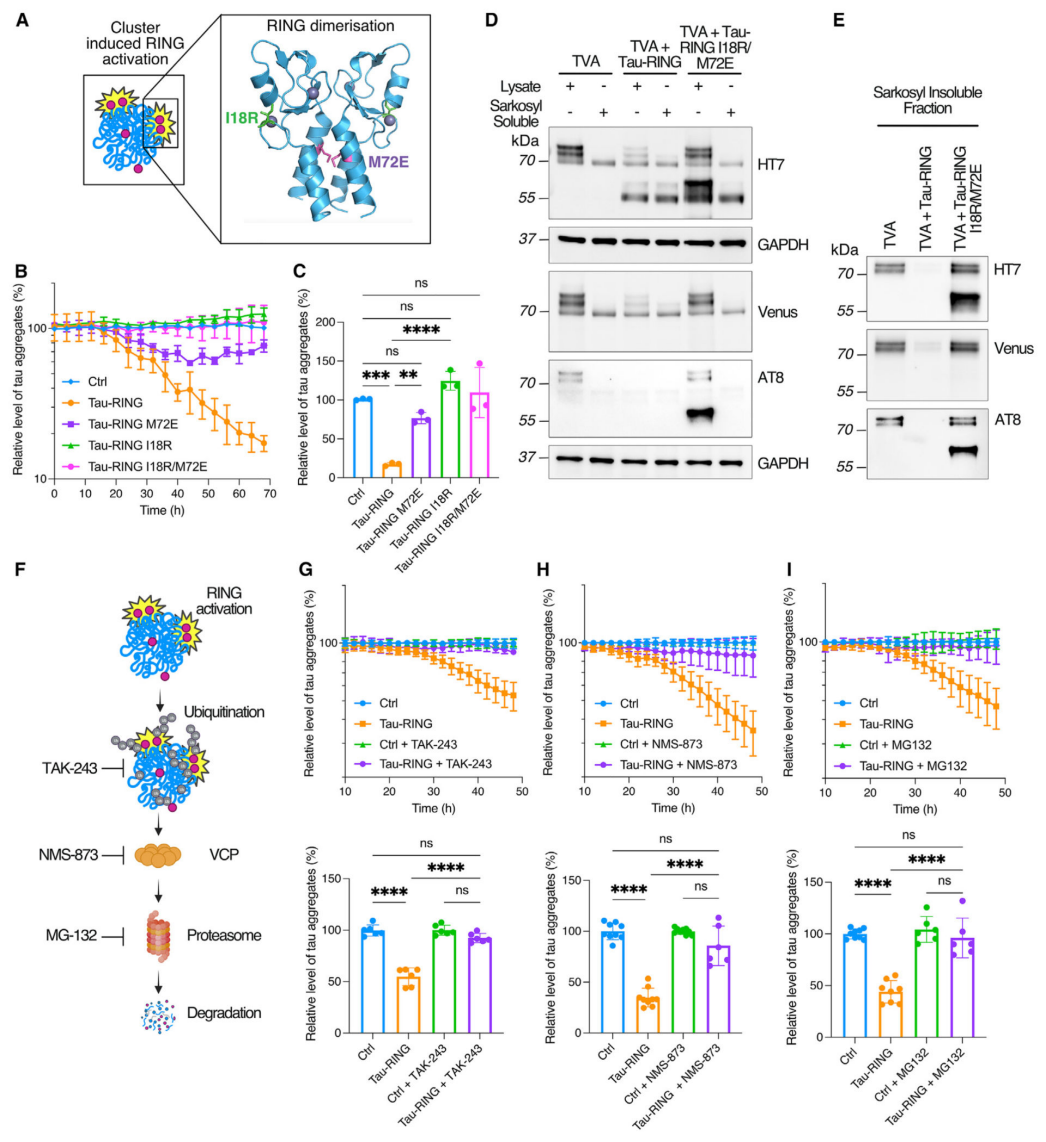
Tau-RING requires canonical TRIM21 pathway components for degradation (A) Schematic of RING domain activation via dimerization. The I18 and M72 residues are highlighted in green and purple, respectively. (B) Time course of tau-RING ± I18R, M72E, I18R/M72E RING mutations in TVA cells. (C) Quantification of the number of aggregates in TVA cells 72 h post transduction with tau-RING lentivirus. *N* = 3. (D and E) TVA cells treated ±tau-RING or tau-RING I18R/M72E lentivirus for 72 h and then fractionated into sarkosyl-soluble and sarkosyl-insoluble tau by ultracentrifugation. Western blots probed for total tau (HT7), venus protein, hyperphorphorylated tau (AT8), and loading control GAPDH. *N* = 3 experiments pooled. (F) Schematic of inhibitors of degradation pathway components utilized by TRIM21. TAK-243 is an E1 inhibitor, NMS-873 is a VCP inhibitor, and MG-132 is a proteasome inhibitor. (G) Time course of TVA cells treated with tau-RING ± TAK-243 (100 nM) and quantification of end point at 48 h post transduction. *N* = 3. (H) Time course of TVA cells treated with tau-RING ± NMS-873 (2 μM) and quantification at 48 h post transduction. *N* = 3. (I) Time course of TVA cells treated with tau-RING ± MG-132 (5 μM) and quantification at 48 h post transduction. *N* = 3. Statistical significance for (C) and (G)–(I) determined by one-way ANOVA and Tukey’s multiple comparisons test. **p < 0.01, ***p < 0.001, ****p < 0.0001; ns, non-significant. See also Figure S3.

The ubiquitination activity of endogenous TRIM21 is activated through substrate-induced clustering, which drives RING dimerization and E2~ubiquitin (Ub) engagement.^15,19^ To determine whether this same clustering mechanism underpins tau-RING function, we introduced two mutations (M72E and I18R) into the RING domain previously shown to prevent RING activity^20^ (Figure 2A). M72E prevents RING dimerization, and I18R renders RING dimers catalytically inactive by inhibiting E2~Ub interaction. We expressed WT, M72E, I18R, or M72E/I18R tau-RING in TVA cells using a lentivirus and monitored degradation over 72 h. All mutations significantly reduced the activity of tau-RING (Figures 2B and 2C). These data support a model where tau-RING becomes incorporated into tau aggregates, stimulating RING multimerization and activation. Upon probing for tau protein, a reduction in tau-venus could be observed in total cell lysate after expressing tau-RING, which correlated with the decrease of tau-venus positive aggregates in the TVA cells observable by live-cell imaging (Figures 2D and S2H). This suggests that the disappearance of tau-venus aggregates is a result of degradation rather than disassembly upon activation of the RING domain. Cells treated for 72 h with lentivirus expressing tau-RING or tau-RING I18R/M72E were also fractionated by ultracentrifugation to observe which species were preferentially degraded by tau-RING. SI and hyperphosphorylated tau were preferentially removed (Figures 2D and 2E).

To test whether tau-RING is dependent upon the ubiquitin-proteasome system (UPS) to degrade aggregates in the same way as endogenous TRIM21, we used previously characterized inhibitors of E1 ubiquitin-activating enzyme (TAK-243), VCP (NMS-873), and the proteasome (MG-132) (Figure 2F).^21–23^ Due to the documented toxicity of these compounds, inhibitors were added 24 h after lentiviral transduction to coincide with tau-RING expression and the initiation of degradation, and cells were evaluated after a further 24 h. E1 inhibitor TAK-243 completely prevented tau-RING-mediated degradation of aggregates in TVA cells, demonstrating that ubiquitination is essential (Figure 2G). Similarly, VCP inhibitor NMS-873 and proteasome inhibitor MG-132 both reversed aggregate removal by tau-RING (Figures 2H and 2I). We also stained TVA cells for poly-Ub chains to investigate if tau-venus aggregates were ubiquitinated upon treatment with tau-RING. 24 h after lentiviral transduction, ubiquitin chains were stabilized by the addition of a deubiquitinating enzyme (DUB) inhibitor (PR-619) for 6 h before staining. Co-localization of tau-venus aggregates with polyubiquitin chains could be observed after treatment with tau-RING (Figure S3A). These data are consistent with previous findings that, after catalyzing the formation of poly-ubiquitin chains, endogenous TRIM21 requires both VCP and the proteasome to inhibit induced tau aggregation by antibody-coated tau seeds.^13^

Autophagic flux is important for maintaining cellular homeostasis, and we therefore wanted to investigate if autophagy remained functional during the degradation of tau aggregates. We therefore probed cells for the ability to increase autophagic flux upon starvation. TVA cells were treated with or without lentivirus expressing tau-RING for 48 h before being starved for 5 h, such that starvation occurred during the peak degradation period. TVA cells were compared with TV cells as a control cell line without aggregates. As expected, upon starvation in all three conditions, p62 was degraded, indicating autophagy was upregulated (Figures S3B and S3C). There was no significant difference in the level of p62 degradation between all three conditions upon induction of starvation, demonstrating the ability of the cells to respond to external stimuli during the degradation process (Figure S3C).

### RING-Bait constructs are effective against seeded aggregation by disease-associated tau aggregates and in neurons

**Figure 3:**
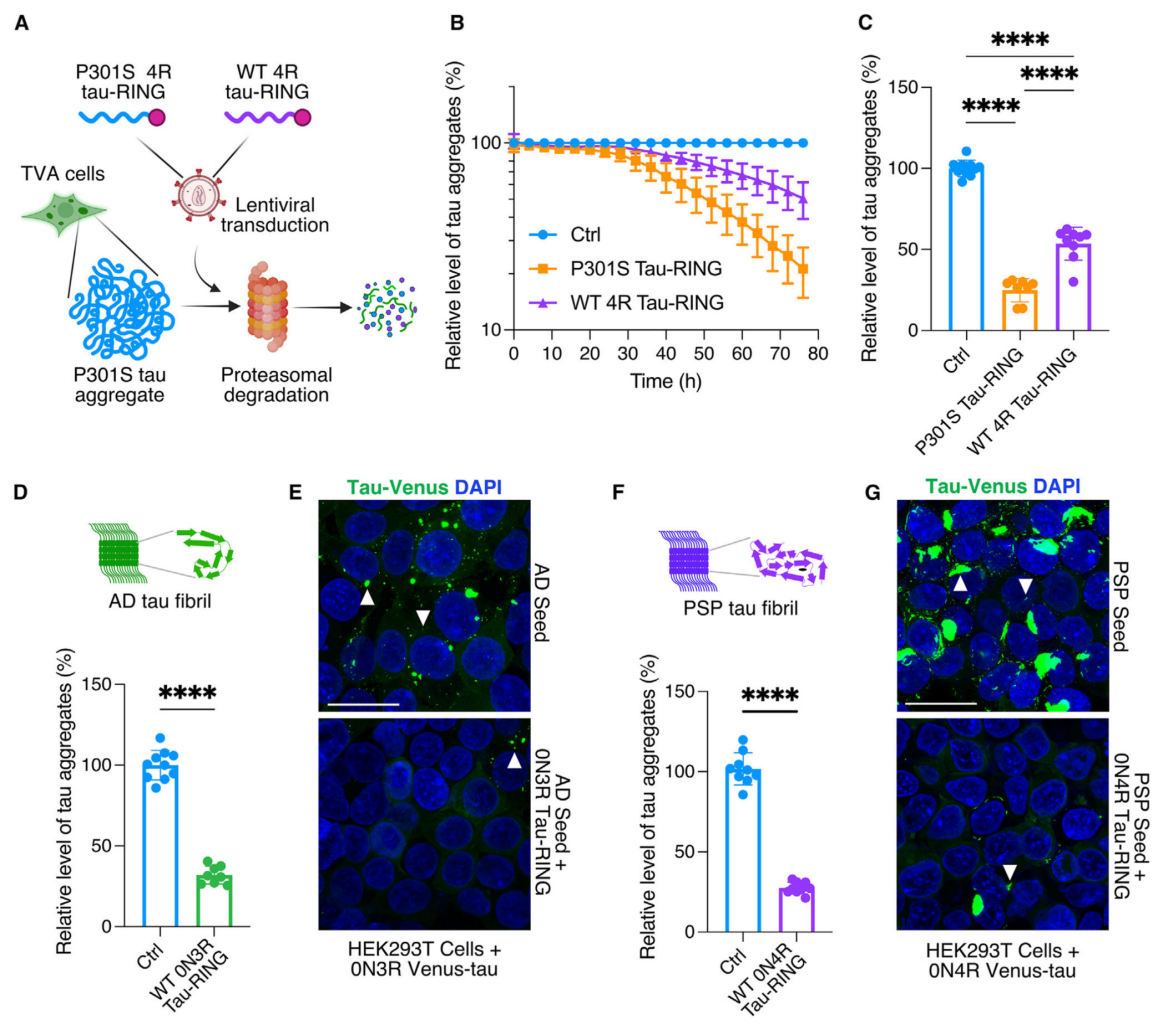
RING-Bait is effective against human brain-derived tau aggregates (A) Schematic of the TVA cell assay, with a lentivirus used to express different isoforms of tau-RING as examples of different “Baits.” Incorporation of the Bait into the aggregate leads to proteasomal degradation and a reduction in the number of puncta by high-content microscopy. (B) Time course of TVA assay, where lentivirus carrying P301S 0N4R tau-RING or WT 0N4R tau-RING is applied to cells. *N* = 3. (C) Quantification of the number of aggregates in TVA cells treated as in (B) 72 h post transduction. *N* = 3. (D) Quantification of the number of aggregates in HEK293T cells expressing venus-3R tau, seeded with AD-derived tau aggregates, ±0N3R tau-RING. *N* = 3. (E) Representative images from cells treated as in (D). (F) Quantification of the number of aggregates in HEK293T cells expressing venus-4R tau, seeded with PSP-derived tau aggregates, ±0N4R tau-RING. *N* = 3. (G) Representative images from cells treated as in (F). White arrows denote tau aggregates. Scale bars, 25 μm. Statistical significance for (C) determined by one-way ANOVA and Tukey’s multiple comparisons test. Statistical significance for (D) and (F) determined by unpaired t test. ****p < 0.0001.

To observe maximum efficiency from a RING-Bait construct, the Bait and substrate may need to be structurally compatible such that the Bait is efficiently incorporated into the target aggregate. For instance, in human tauopathies, the filaments in different diseases are composed of different tau isoforms.^24,25^ In familial tauopathies, incorporation of WT tau vs. mutant tau into filaments is mutation dependent.^25^ We therefore explored the efficacy of WT 0N4R tau-RING in removing pre-existing aggregates in TVA cells, which carry the P301S mutation. WT 0N4R tau-RING was less effective than P301S 0N4R tau-RING at degrading aggregates in TVA cells (Figures 3A–3C). As expected, this indicates that amyloid folds of tau bearing the P301S mutation are less efficient at templating WT tau.

The fold of tau in heparin-assembled filaments is known to be heterogeneous and different to all resolved structures from human tauopathies.^26^ The fold adopted in the TVA cells is unknown, but also likely to be different from human disease conformations. To show that the RING-Bait strategy is not limited to a particular fold or aggregate structure, we tested the ability of WT tau-RING constructs to intercept aggregation induced by AD and PSP post-mortem brain extracts. SI tau was isolated from AD brains and applied to HEK293T cells expressing WT 0N3R venus-tau, as it has recently been shown AD tau aggregates preferentially seed WT 3R tau over 4R tau.^27,28^ After the appearance of venus puncta, lentivirus was used to deliver WT 0N3R tau-RING. The number of aggregates was then assessed 72 h later. WT 0N3R tau-RING significantly reduced the number of aggregates seeded from AD-derived SI tau (Figures 3D and 3E). A parallel system was set up with WT 0N4R venus-tau to use with PSP-derived SI tau aggregates, as PSP is a 4R tauopathy. 0N4R tau-RING was also effective at removing aggregates seeded from PSP SI tau (Figures 3F and 3G). These data suggest that a RING-Bait approach can be used to prevent seeded aggregation from known human tauopathy folds.

**Figure 4:**
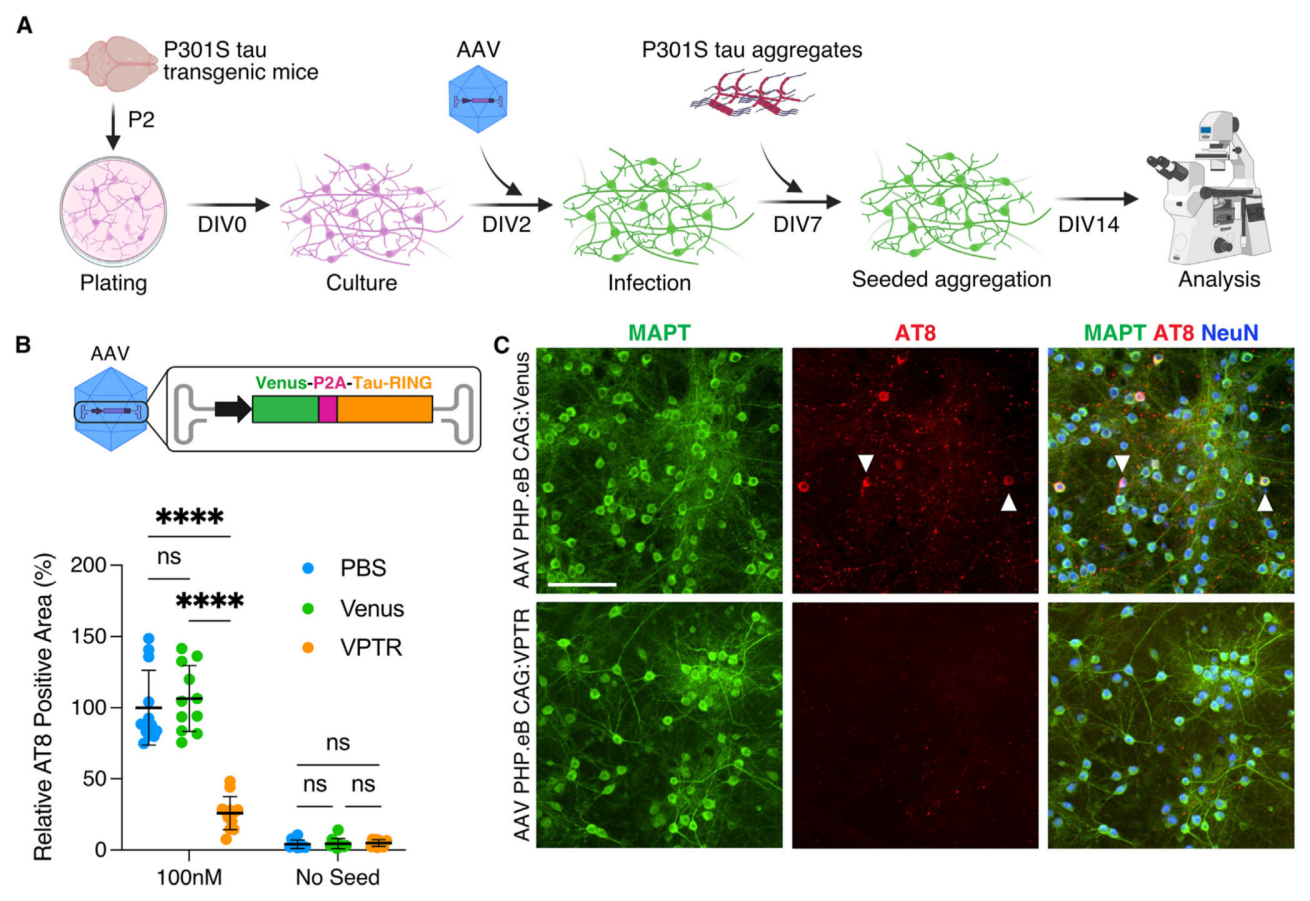
Tau-RING prevents seeded aggregation in primary neurons (A) Schematic of the primary neuron seeding assay, using P301S tau transgenic mice at P2. Cultures were infected with AAV PHP.eB carrying venus-P2A-tau-RING (VPTR) or venus only at day *in vitro* 2 (DIV2), and P301S tau aggregates were added to the media at DIV7. Cultures were evaluated at DIV14 for the number of AT8-positive tau aggregates. (B) Quantification of primary neurons treated ±100 nM tau aggregates, ±venus, or venus-P2A-tau-RING (VPTR) AAV at DIV14. *N* = 3. (C) Representative immunofluorescence images of primary neuron cultures at DIV14, treated with venus or VPTR AAV, +100 nM P301S tau aggregates. Scale bar, 100 μm. Statistical significance for (B) determined by two-way ANOVA and Sidak’s multiple comparisons test. ****p < 0.0001. ns, non-significant. See also Figure S3.

TV cells are a useful model to study tau aggregation; however, as HEK293Ts are dividing cells, they differ significantly to postmitotic cells such as neurons with regards to protein turnover. To validate the efficacy of RING-Bait in a neuronal setting, we generated a recombinant adeno-associated virus (AAV) encoding the gene for fluorescent protein venus to allow easy visualization, followed by a P2A co-translational self-cleaving peptide and tau-RING. Ribosomal translation of venus-P2A-tau-RING (VPTR) mRNA produced the expected cleavage products of venus, tau-RING, and a small amount of full-length VPTR when expressed in HEK cells (Figure S3D). We introduced this cassette into an AAV with an engineered capsid (PHP.eB) that preferentially infects neurons and is reported to cross the blood-brain barrier (BBB) independently in mice (Figure S3E).^29^ Primary neurons from transgenic mice expressing P301S tau were infected with AAV PHP.eB carrying venus or VPTR at day *in vitro* (DIV) 2 (Figure 4A). As in HEK cells, the expression of tau-RING in neurons did not reduce levels of native soluble tau (Figures S3F and S3G). P301S tau aggregates pre-assembled with heparin were added to the media at DIV7, and aggregation was analyzed at DIV14 by AT8 staining (Figure 4A). Expression of AAV-delivered tau-RING resulted in a significant decrease in seeded aggregation of approximately 75%, quantified by AT8-positive area (Figures 4B and 4C). Tau-RING almost completely prevented the accumulation of AT8-positive aggregates in cell bodies (Figure S3H). A substantial reduction of aggregates in neuronal processes was also observed, though of lower magnitude (Figure S3I). Primary cultures were stained for neuron-specific antigen NeuN to determine if there was any cell death caused by either the delivery of the construct or its activation upon the addition of tau aggregates. We observed a reduction in cell count in all conditions upon the addition of tau aggregates; however, there was no independent cell death caused by the delivery of the viral constructs or the degradation process initiated by tau-RING (Figure S3J). These data demonstrate that RING-Bait constructs are effective at preventing the seeded aggregation of tau in primary neurons without overt toxicity.

### RING-Bait technology can reduce tau pathology *in vivo*

**Figure 5:**
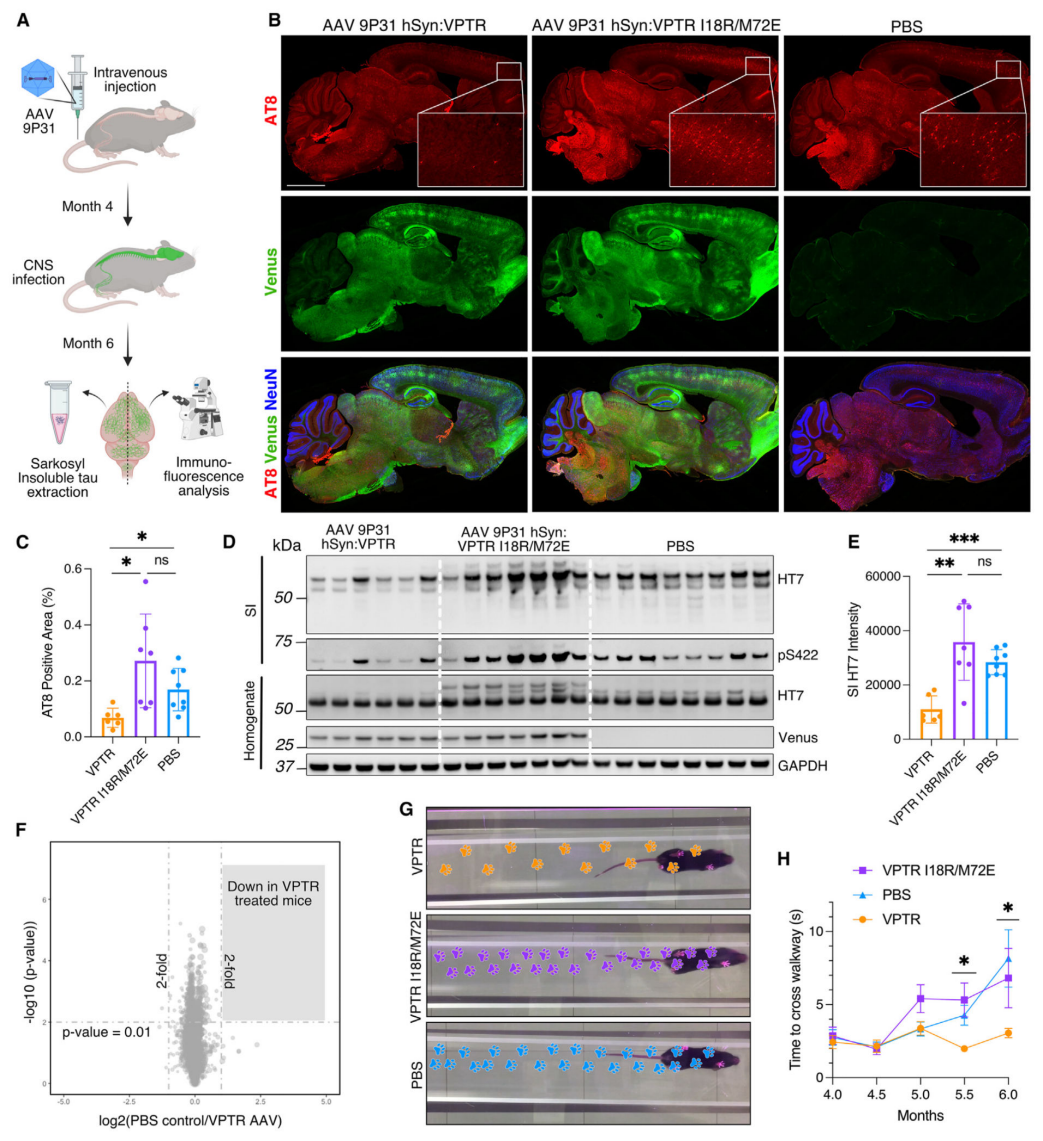
Tau-RING reduces tau pathology *in vivo* and improves motor function (A) Schematic of intravenous injection of P301S mice at 4 months with AAV 9P31. At 6 months, one half of the brain was homogenized in order to extract sarkosyl insoluble (SI) tau assemblies, and the other half was fixed and analyzed for AT8-positive tau aggregates by immunofluorescent staining. (B) Representative immunoflourescence images of mice infected with AAV 9P31 hSyn:VPTR (active tau-RING) or hSyn:VPTR I18R/M72E (inactive tau-RING) at 4 months, or injected with PBS, and evaluated at 6 months for tau aggregates via AT8 staining. Venus fluorescence detected from virally expressed protein. Neuronal nuclei were probed for using an antibody against NeuN. Enlarged cortical region shown to exemplify tau aggregate levels. Scale bar, 2 mm. (C) Quantification of AT8-positive tau aggregates in frontal cortex, as shown in (B). (D) Western blot of the sarkosyl insoluble (SI) fraction of mouse brains treated as in (B), stained for total human tau (HT7) and hyperphosphorylated tau at serine 422 (pS422). Mouse brain homogenate was also probed with HT7 and venus protein, in addition to GAPDH as a loading control. (E) Quantification of SI HT7 in (D). (F) Mass spectrometry analysis of whole brain homogenate from mice treated with PBS compared with hSyn:VPTR AAV. (G) Footsteps of the median mouse from each condition (VPTR, VPTR I18R/M72E, PBS) on the MouseWalker apparatus. (H) Quantification of the time to traverse the walkway for mice from each condition (VPTR, VPTR I18R/M72E, PBS) from 4 to 6 months. *N* = 6–8 mice per group. Statistical significance for (C) and (E) determined by Brown-Forsythe and Welch ANOVA. Statistical significance for (F) determined by unpaired t test with group-wise correction. Statistical significance for (H) determined by Kruskal-Wallis test. *p < 0.05. **p < 0.01. ****p < 0.0001. ns, non-significant. See also Figures S4 and S5.

Having determined that tau-RING was effective at reducing seeded aggregation in primary neurons, we delivered tau-RING to an animal disease model of tauopathy. Tg2541 mice express P301S tau driven by the Thy1 promoter and develop tau pathology predominantly in the spinal cord, brain stem, and frontal cortex.^30^ Pathology in these areas is generally well-developed at 6 months, and mice begin to display motor symptoms; therefore, this time point was chosen as the endpoint for all experiments. AAV of the same serotype used in the primary neurons, PHP.eB, was produced and delivered systemically to the mice. However, this capsid poorly transduced the brain in Tg2541 mice. We therefore explored the use of another recently generated AAV capsid, 9P31.^31^ VPTR, with or without the inactivating RING mutations I18R/M72E, was packaged in AAV 9P31 under the human synapsin promoter and injected into the tail vein of P301S mice at 4 months of age. Tau pathology was evaluated at 6 months. Upon study completion, one hemisphere from each mouse was fixed and cryo-sectioned. The other half was processed for western blot, including the SI extraction of tau (Figure 5A).

In the fixed samples, tau aggregates were probed for using the antibody AT8 (Figure 5B). Delivery of VPTR AAV resulted in a significant decrease in AT8-positive aggregates in the frontal cortex compared with VPTR I18R/M72E-treated mice or PBS-treated control mice (Figure 5C). AT8 aggregates were increased in the VPTR I18R/M72E-treated mice, although not significantly compared with the PBS control. SI tau was extracted from one hemisphere of each brain and visualized by western blot (Figure 5D). A significant decrease in total SI tau, probed for with the antibody HT7, was observed in mice injected with VPTR compared with VPTR I18R/M72E-treated mice or PBS-treated control mice (Figures 5E and S4A). To evaluate the reduction in tau aggregates achieved through treatment, relative to the starting time point of 4 months, SI tau levels were compared with those from 4-month-old P301S mice. No difference in homogenate levels of total tau was observed between 4-month-old P301S mice and 6-month-old mice treated with VPTR AAV or PBS; however, higher molecular weight tau bands were absent at this time point (Figures S4B and S4C). A significant increase in SI tau was observed in PBS-treated mice at 6 months compared with those at 4 months (Figures S4D and S4E). SI tau levels in VPTR-treated mice at 6 months were not significantly higher than at 4 months but significantly lower than those in PBS-treated 6-month-old mice (Figure S4E). This suggests that treatment effectively slows down the progression of tau pathology *in vivo*. In addition to total tau, levels of hyperphosphorylated tau in the SI fraction were reduced in VPTR-treated mice compared with controls (Figures 5D and S4F). In the total brain homogenate, higher molecular weight species of tau above the main band (~50 kDa) were significantly reduced in the VPTR-treated group, confirming this observation (Figures 5D and S4G). Homogenate levels of tau at ~50 kDa were not significantly different between groups (Figures 5D and S4H). Whole brain homogenate was probed for venus protein, which demonstrated transduction of the brain was consistent between the VPTR- and VPTR I18R/M72E AAV 9P31-treated mice (Figure S4I). Tau-RING was therefore able to significantly and selectively reduce the burden of tau aggregates *in vivo*.

To evaluate whether tau-RING-mediated degradation of tau aggregates resulted in off-target degradation of non-specific proteins, we carried out mass spectrometry analysis of total brain homogenate from the mice treated in Figure 5D. Over 8,000 protein groups were quantified with no off-target degradation effects observed (*p* < 0.01, fold change > 2) when comparing all three groups (Figures 5F, S5A, and S5B). We further validated the mass spectrometry data by evaluating the levels of neuron-specific β-III tubulin, a prevalent tau-binding partner. The levels of β-III tubulin remained consistent across all treatment groups both by mass spectrometry and western blot analysis, providing additional evidence that tau-RING does not lead to off-target degradation of important binding partners such as microtubule proteins (Figures S5C–S5E).

As there is variation in tau pathology between P301S mice, we carried out a further study in which we stereotaxically injected tau-RING into one hemisphere, enabling the quantification of the number of tau aggregates to be controlled within each mouse. This also allowed us to investigate whether tau-RING could remove pre-existing aggregates in older mice. Mice were injected in the frontal cortex at 5 months old with VPTR or venus only AAV PHP.eB and culled 4 weeks later (Figure S5F). Injected hemispheres were compared with the contralateral, uninjected hemisphere. Comparison of individual injected vs. contralateral hemispheres showed a consistent decrease in AT8 area in all tau-RING-treated mice in comparison to venus-only-treated mice (Figures S5G and S5H). These data provide further support that the expression of tau-RING leads to a significant and substantial reduction in tau pathology.

To investigate whether a reduction in tau pathology correlated with an improvement in motor function in P301S mice, we constructed a MouseWalker that allows motor behavior to be assessed non-invasively.^32^ Videos of the mice were collected every 2 weeks, from 4 to 6 months, and analyzed by using open-source software provided by DeepLabCut.^33^ DeepLabCut was trained to identify footprints as the mice traversed the MouseWalker platform. Measures including the number of footsteps and time in contact with the platform could be extrapolated. Treatment with VPTR resulted in a significant improvement in hind leg use and prevented the decline in time to cross the walkway observed in comparison to mock-treated animals (Figures 5G and 5H; Figures S5I–S5L). The reduction in pathology observed upon delivery of tau-RING therefore resulted in measurable benefits in the motor phenotype of P301S mice.

## Discussion

Intracellular protein aggregates are thought to drive many neuro-degenerative diseases, but there are currently no approved therapies that target them. Future therapies should act in the intracellular compartment and preferentially target the assembled species, sparing functional copies of the protein. We have developed RING-Bait technology to provide a strategy for targeting intracellular aggregates for degradation. RING-Bait comprises two components: a Bait element whose sequence matches at least part of the protein aggregate to be targeted and a RING domain from the E3 ligase TRIM21 that is activated by substrate-induced clustering. In cells where aggregates are forming, RING-Bait is incorporated into growing assemblies along with other monomeric copies of the aggregating protein. The incorporation of multiple RING-Baits into a large repetitive structure creates the conditions for RING activation, resulting in ubiquitination and degradation of the aggregate while crucially leaving monomeric protein copies intact.

We have exemplified RING-Bait technology by using it to target tau aggregates for degradation. We show that expression of tau-RING successfully induces the removal of assembled but not monomeric tau in HEK293 cells, primary neurons, and *in vivo*. Efficient aggregate degradation was dependent upon RING activity, the ubiquitin-selective segregase p97/VCP, and the proteasome. It has recently been postulated that VCP and the proteasome incompletely disassemble tau aggregates, fragmenting them into smaller seed-competent species.^34,35^ However, we found that tau-RING-mediated degradation resulted in the complete clearance of aggregates, leaving behind very few seed-competent species. One possible reason for this high level of clearance is that tau-RING can work iteratively, incorporating itself into any new seeds generated by the proteasome and ensuring they are continuously re-targeted for degradation. It has also been reported that tau aggregates themselves can inhibit the proteasome,^36^ which is itself associated with significant toxicity. We did not observe cellular toxicity associated with the degradation of tau aggregates, as cells bearing aggregates and treated with a RING-Bait construct continued to be viable. Additionally, when treated cells were stressed with starvation, autophagic flux was upregulated as expected. Therefore the two main cellular degradation pathways remained functional during our treatment.

It has been reported that tau aggregates can persist for long periods of time within cells, but also that they are dynamic structures that are continuously turning over.^37^ Our data support previous findings that tau aggregates are not “tombstones,” as the incorporation of new material is necessary for tau-RING to clear pre-existing aggregates in our model systems. Interesting differences were observed in the capacity for primary neurons to prevent aggregation in processes vs. cell bodies, with more efficient protection observed against tau accumulation in cell bodies. Tau assemblies have been shown to transfer between synaptically connected neurons to induce seeded aggregation.^38,39^ Tau aggregates traveling between cells are therefore likely to enter cells within a process and consequently may be less efficiently cleared. Further exploration of this potential vulnerability is warranted.

A significant challenge facing protein-based therapeutics is how to deliver them into neurons. Here, we have used a gene therapy approach, where a BBB-crossing AAV delivers RING-Bait cargo directly into the mouse brain via intravenous injection. We initially experimented with AAV PHP.eB^29^; however, this did not efficiently transduce the brain in Tg2541 mice. The AAV capsid 9P31 was able to efficiently cross the BBB in these mice, and we observed a significant reduction in tau pathology, both through immunofluorescence analysis of AT8-positive tau aggregates and in the SI fraction of tau in brain homogenate.

A further challenge for anti-aggregate therapies is achieving not just aggregate removal but also a concomitant benefit in phenotypic outcome. To investigate this, we established a MouseWalker system so that we could correlate aggregate removal with changes in motor function.^32^ Using this system, we were able to demonstrate a significant improvement in the motor phenotype of tau-RING-treated mice, as assessed by multiple measures including both fine motor control (number of footsteps) and speed of movement (time to traverse walkway). These data provide some encouragement that the level of targeted tau aggregate degradation by RING-Bait technology is sufficient to achieve observable benefits to mouse motor function. However, future work will be needed to demonstrate benefit in other models of tauopathy and to refine delivery to achieve optimal levels of transduction.

While we have exemplified RING-Bait in the context of tau aggregates, we believe the technology could be applied widely to other neurodegenerative aggregate-based diseases. The underlying concept of using a component part of the aggregate in order to target it provides a targeting approach that obviates the need for specific binders, such as antibodies, to be made against each neurodegenerative aggregate. Given the growing evidence that the same proteins can form distinct structures in different diseases, potentially requiring specific binders to be developed against each, this is a significant advantage. Importantly, RING-Bait not only ensures specific aggregate targeting but simultaneously uses incorporation as the trigger that activates RING E3 function and recruits cellular degradation machinery. This is possible because of the particular ubiquitination mechanism of the TRIM21 RING, which is only activated when its RING domain undergoes substrate-induced clustering, i.e., when multiple copies of the RING are brought into close proximity.^15^ Other RING domains may be capable of similar function, for instance from TRIM5.^40^ Overall, we believe this technology provides an effective and specific method for removing neurodegenerative protein aggregates and is a promising therapeutic strategy.

### Limitations of the study

In this study, we have detailed how tau aggregates can be targeted for proteasomal degradation using a RING-Bait therapeutic. While we have provided evidence that a reduction in tau aggregates leads to an improvement in mouse motor phenotype, further work will be required to correlate a reduction in tau aggregates with the survival of individual neurons or an improvement in their function. In our study, we were unable to evaluate neuron survival in the spinal cords of the mice due to our tissue collection and processing methods. This will be important to evaluate in future work. We have also not determined the precise concentration of tau-RING needed to achieve efficient degradation at different levels of tau expression and abundance of tau aggregates. Future research could investigate this using inducible promoter systems. This information would be useful for further therapeutic development, in addition to assessing the necessary viral titer for treatment administration and promoter strength required for optimal RING-Bait expression. It is not known if the AAV 9P31 capsid used in this study can cross the human BBB; however, previous capsids developed for the mouse have not retained this ability.^41^ Therefore, a limitation of this study is that a different AAV may be necessary to deliver RING-Bait in a clinical setting. There has been significant progress in developing capsids that are BBB-penetrant in non-human primates (NHPs).^42,43^ Future work will also be needed to validate mitigation strategies against the potential for an immune response after systemic delivery in adult patients with prior exposure to AAV.^44^ Intrathecal and stereotaxic delivery routes are additionally being investigated for a range of CNS therapeutics, which could circumvent this problem.^45,46^ Despite their challenges, one advantage of AAVs is that they persist in an episomal state within human cells for extended periods. Given our target is a non-dividing cell population, therapy via this route may be effective with a single administration, thereby mitigating the risk of AAV-specific antibodies being generated during a multi-dosing regime.

## Star⋆Methods

### Key Resources Table

**Table:** 

REAGENT or RESOURCE	SOURCE	IDENTIFIER
Antibodies		
AT8 (1:250)	ThermoFisher	Cat# MN1020
AT8-Biotin (1:1000)	ThermoFisher	Cat# MN1020B
pS422 (1:400)	ThermoFisher	Cat# 44-764-G
HT7 (1:1000)	ThermoFisher	Cat# MN1000
Pan-tau (1:2000)	DAKO	Cat# A0024
NeuN (1:500)	Abcam	Cat# Ab177487
FK2 (1:500)	Merck	Cat# 04-263
GAPDH (1:2000)	Cell Signaling Technology	Cat# 14C10
CypB (1:500)	Santa Cruz Biotechnology	Cat# Sc-130626
β-actin (1:2000)	Santa Cruz Biotechnology	Cat# Sc-47778
β-III-tubulin (1:500)	Abcam	Cat# Ab78078
P62 (1:1000)	ThermoFisher	Cat# PA5-20839
MAP2 (1:1000)	Abcam	Cat# Ab5392
GFP (Venus) – (1:1000)	Proteintech	Cat# 50430-2-AP
Anti-rabbit Alexa 488 (1:1000)	ThermoFisher	Cat# A-11008
Anti-rabbit Alexa 568 (1:1000)	ThermoFisher	Cat# A-11011
Anti-rabbit Alexa 647 (1:1000)	ThermoFisher	Cat# A-21245
Anti-mouse Alexa 488 (1:1000)	ThermoFisher	Cat# A-11001
Anti-mouse Alexa 568 (1:1000)	ThermoFisher	Cat# A-11031
Anti-mouse Alexa 647 (1:1000)	ThermoFisher	Cat# A-21235
Streptavidin Alexa 647 (1:1000)	ThermoFisher	Cat# S32357
Anti-mouse-HRP	ThermoFisher	Cat# A16066
Anti-rabbit-HRP	ThermoFisher	Cat# 65-6120
Bacterial and virus strains		
BL-21 (DE3)	Agilent Technologies	Cat# 200131
Rosetta 2 DE3	Novagen	Cat# 71397
XL Gold Ultracompetent Cells	Agilent	Cat# 200314
AAV PHP.eB	Addgene	Cat# 103005
AAV 9P31	Nonnenmacher et al.^31^	N/A
Chemicals, peptides, and recombinant proteins		
T4455	Sigma-Aldrich	Cat# T4455
MG132	Sigma-Aldrich	Cat# M7449
NMS-873	Selleckchem	Cat# S7285
TAK-243	Selleckchem	Cat# S8341
PR-619	Sigma-Aldrich	Cat# 662141
Bafilomycin	Selleckchem	Cat#S1413
0N4R P301S tau	This paper	N/A
0N4R P301S tau-RING	This paper	N/A
Biological samples		
AD human brain	Oxford Brain Bank	N/A
PSP human brain	Cambridge Brain Bank	N/A
Deposited data		
Bulk mass spectrometry data	PRIDE	PXD052897
Experimental models: Cell lines		
HEK293T	ATCC	Cat# CRL-3216
TV cells	McEwan et al.^13^	N/A
TVA cells	This paper	N/A
BHK cells	ATCC	Cat# [C-13]-CCL-10
Experimental models: Organisms/strains		
Thy1-hTau.P301S mice (CBA.C57BL/6)	M. Goedert laboratory Allen et al.^30^	Mouse strain: Tg2541
Recombinant DNA		
pUCmini-iCAP-PHP.eB	Addgene	Cat# 103005
pAAV-CAG-GFP	Addgene	Cat# 37825
pAdDeltaF6	Addgene	Cat# 112867
pMDG2	Addgene	Cat# 12259
pCRV GagPol	Mallery et al.^47^	N/A
pSMPPv2-Tau-RING	This paper	N/A
pSMPP-Tau-mCherry	This paper	N/A
pSMPPv2-Tau-mCherry-RING	This paper	N/A
pSMPPv2-Nanobody-mCherry-RING	This paper	N/A
pSMPPv2-Tau-RING (M72E/I18R)	This paper	N/A
pOPTG-Tau-RING	This paper	N/A
pOPTG-Tau-RING (M72E/I18R)	This paper	N/A
AAV 9P31-hSyn-Venus-P2A-Tau-RING	This paper	N/A
AAV 9P31-hSyn-Venus-P2A-Tau-RING I18R/M72E	This paper	N/A
AAV PHP.eB-CAG-Venus	This paper	N/A
AAV PHP.eB-CAG-Venus-P2A-Tau-RING	This paper	N/A
Software and algorithms		
DeepLabCut	Mathis et al.^33^	https://github.com/DeepLabCut
ImageJ	Schindelin et al.^48^	https://fiji.sc
BioRender	BioRender	https://biorender.com
Prism 10	Graphpad	https://www.graphpad.com
Other		
HisTrap FF	GE Healthcare	Cat# 17-5255-01
HisTrap HP	GE Healthcare	Cat# 17-5248-01
HiLoad 16/600 Superdex 200	GE Healthcare	Cat# 28-9893-35
Amicon Ultra-15 concentrators	Merck Millipore	Cat# UFC901024
Amicon Ultra-100 concentrators	Merck Millipore	Cat# UFC910008
Lipofectmine 2000	ThermoFisher	Cat# 11-668-027
OptiMEM	Gibco	Cat# 31985062
DMEM	ThermoFisher	Cat# 11995065
MES Buffer	ThermoFisher	Cat# B0002
MOPS Buffer	ThermoFisher	Cat# J62847-AP
NuPAGE LDS sample buffer	Invitrogen	Cat# NP0008
4X NuPAGE BisTris gel	Invitrogen	Cat# NP0323BOX
FuGENE6	Promega	Cat# E2691
ECL substrate	Merck	Cat# WBKLS0500
Precision Plus Protein Kaleidoscope Ladder	BioRad	Cat# 1610395
PVDF membrane	BioRad	Cat# 1704272
CLARIOstar	BMG Labtech	CLARIOstar
NuPAGE Bis-Tris gel	Invitrogen	Cat# NP0323BOX
Protein kaleidoscope Ladder	BioRad	Cat# 1610395
Prolong Diamond Antifade Mountant	ThermoFisher	Cat# P36961
Glass coverslips	ThermoFisher	Cat# 18X18-1
Carbon Film Supported Copper Square Mesh	Merck	Cat# TEM-CF400-CU-TH
OCT	Sakura	Cat# 94-4583
Glycerol	ThermoFisher	Cat# 15514029
Ethylene Glycol	ThermoFisher	Cat# 29810
Iodixanol	Sigma	Cat# D1556-250
Benzonase	Sigma	Cat# E1014-25KU
Phenol Red	Sigma	Cat# P0290
QIAGEN Plasmid Mini Kit	QIAGEN	Cat# 12123
QIAGEN Plasmid Midi Kit	QIAGEN	Cat# 12143
QIAGEN Plasmid Maxi Kit	QIAGEN	Cat# 12162
PEG8000	VWR	Cat# 71003-444
PEI	Generon	Cat# 24765-1
Optiseal Beckman tubes	ThermoFisher	Cat# 362183
Beckman 1.5 ml Polypropylene Tubes	Beckman Coulter	Cat# 356094
Goat Serum	ThermoFisher	Cat# 16210064
Fetal Bovine Serum	ThermoFisher	Cat# A5670701
Horse Serum	Invitrogen	Cat# 26050070
Paraformaldehyde	ThermoFisher	Cat# 047392.9M
Poly-D-Lysine	Invitrogen	Cat# A3890401
Poly-L-Lysine	RnD Systems	Cat# 3438-100-01
Neurobasal Plus	Gibco	Cat# A3582901
B-27 plus 50x supplement	Gibco	Cat# A35828-01
100x Glutamax	Invitrogen	Cat# 35050038
1% DNAse	Sigma	Cat# DN25-100MG
Hibernate-A	ThermoFisher	Cat# A1247501
Penicillin/Streptomycin	Invitrogen	Cat# 15140122
Heparin	Sigma	Cat# H0878
DTT	Merck	Cat# 10197777001
Thioflavin S	Sigma	Cat# T1892
Halt Protease and Phosphatase inhibitor	ThermoFisher	Cat# 78440

### Experimental Model and Study Participant Details

#### Mouse lines

All animal work was licensed under the UK Animals (Scientific Procedures) Act 1986 and approved by the Medical Research Council Animal Welfare and Ethical Review Body. P301S tau transgenic mice that had been extensively backcrossed to C57BL/6 background were obtained from Prof Michel Goedert, Cambridge LMB (Tg2541). Animals were regularly monitored for clinical signs for the duration of all experiments. Animals that displayed clinical signs which did not improve within a 6-hour period were sacrificed. Male and female mice were used equally in this study. Mice were randomised into groups by sex and age for *in vivo* experiments. Experiments utilised mice from 4-6 months of age. Male and female mouse brain tissue (postnatal day 2) was pooled for the preparation of primary neuron cultures.

#### Cell lines

HEK293T (ATCC; CRL-3216 - female), HEK293 (ATCC; CRL-1573 - female) and BHK-21 (ATCC; [C-13] - CCL-10 - male) cells were used in this study. All cells were cultured in DMEM medium (Gibco; 31966021) supplemented with 10% fetal bovine serum (FBS) and 5 mg/ml penicillin-streptomycin (P/S). All cells were grown at 37 °C in a 5% CO_2_ humidified atmosphere and regularly checked for mycoplasma. 0N4R P301S tau-venus HEK293 cells (TV cells) were used from a previous publication.^13^ TV cells expressing tau-ring were generated using a lentivirus encoding for the gene, and then selection with puromycin was carried out to obtain resistant cells. TVA cells (TV cells constitutively bearing aggregates) were created by seeding TV cells with Sarkosyl Insoluble extract from aged P301S mice and selecting for a clone which stably propagated aggregates.

### Method Details

#### Plasmids

Plasmids coding for Tau-RING, Tau-RING(M72E/I18R), Tau-mCherry-RING for mammalian cell expression were obtained from a geneBlock (IDT) containing Tau-RING, Tau-RING (M72E/I18R) and Tau-mCherry-RING and inserted in the pSMPPv2 vector using Gibson assembly. pAAV-Venus-P2A-Tau-RING was obtained by cloning Venus-P2A-Tau-RING into the pAAV-CAG-EGFP. Plasmids used in the study are listed in the key resources table.

#### Lentivirus production

VSV HIV-1 pseudotypes were produced as previously described.^47^ Briefly, 2.5 × 10^6^ cells were plated in a 10 cm dish the day before. Transfection mixtures were made using 200 μl of Opti-MEM (Gibco), 1 μg of pMDG2, 1 μg of pCRV GagPol, 1.5 μg of the respective pSMPPv2 plasmids encoding Tau-RING versions, and 12 μl of FuGENE6 (Promega). Mixtures were incubated at room temperature for 20 mins and then added to the 10 cm dishes. Viral supernatants were harvested 48 hrs after transfection, filtered through a 0.45 μm filter and stored at −80°C. For some experiments, the particles were concentrated by ultracentrifugation over a 20% (w/v) sucrose cushion (3 hrs at 28,000 rpm in a Beckman SW32 rotor, Beckman Coulter Life Sciences). The pellet was resuspended in PBS and incubated at 4°C for 6 hrs to allow for full resuspension.

#### Lentivirus quantification

Lentivirus was quantified by the level of RT enzyme using quantitative reverse transcription polymerase chain reaction (PCR). Briefly, 5 μl of viral supernatant was mixed with 5 μl of lysis buffer (0.25% Triton X-100, 50 mM KCl, 100 mM tris–HCl (pH 7.4) and 40% glycerol) and 0.1 μl of ribonuclease (RNase) inhibitor and incubated for 10 mins at room temperature before diluting to 100 μl with nuclease-free water. Two microliters of lysate were added to 5 μl of TaqMan Fast Universal PCR Mix, 0.1 μl of MS2 RNA, 0.05 μl of RNase inhibitor and 0.5 μl of MS2 primer mix, to a final volume of 10 μl. The reaction was run on an ABI StepOnePlus Real-Time PCR System (Life Technologies), with the additional reverse transcription step (42°C for 20 min).

#### Recombinant tau production

The expression and purification of recombinant human 6xHis-0N4R tau bearing the P301S mutation from *E. coli* BL-21 (DE3, Agilent Technologies) was performed as described previously.^16^ Bacterial pellets were collected through centrifugation (3300 g, 4 °C, 10 min) and then resuspended in 10 ml/L of culture with buffer A (25 mM HEPES, pH 7.4, 300 mM NaCl, 20 mM Imidazole, 1 mM benzamidine, 1 mM PMSF, 14 mM β-mercaptoethanol, 1% NP-40, 1 x complete EDTA-free protease inhibitors). The resuspended bacteria were lysed on ice using a probe sonicator and boiled for 10 min at 95 °C. Denatured proteins were pelleted by ultracentrifugation at 100,000 g, 4°C for 50 mins. The clarified supernatant containing 6xHis-tagged monomeric P301S tau with a TEV site was then passed through a HisTrap FF column according to manufacturer instructions (GE Healthcare). Fractions of interest were concentrated using 10 kDa cut-off Amicon Ultra-15 concentrators (Merck Millipore). To remove the 6xHis tag, tau was incubated with TEV protease following manufacturer’s instructions (Sigma-Aldrich, T4455). After incubation, the pooled fractions were loaded onto a second HisTrap HP column to remove the protease and the successfully cleaved tau was collected. Cleaved tau was concentrated using a 10 kDa cut-off Amicon Ultra-15 concentrator (Merck Millipore) before loading onto a HiLoad 16/600 Superdex 200 (Cytiva) size exclusion chromatography column. All purification was performed on an ÄKTA Pure system (Cytiva). Purified tau was concentrated to at least 3 mg/mL using a 10 kDa cut off Amicon Ultra-15 concentrator (Merck Millipore) and snap-frozen in liquid nitrogen for storage at -80 °C in PBS containing 1 mM DTT.

#### Recombinant tau aggregation

Tau monomer was added to Tau Aggregation Buffer (20 μM heparin, 60 μM P301S tau monomer, 2 mM DTT, 1X protease inhibitor, 1X PBS) and incubated at 37°C for 3 days. The resulting P301S tau filaments were sonicated for 15 seconds before long-term storage at -80°C. To observe the kinetics of the reaction, tau monomer was added to Tau Aggregation Kinetics Buffer (2.5 μM heparin, 7.5 μM P301S tau monomer, 2 mM DTT, 1X protease inhibitor, 10 μM Thioflavin S, 1X PBS) and samples were loaded in triplicate into black 96-well plates. Plates were loaded into a CLARIOstar (BMG Labtech), and measurements were taken every 5 minutes after shaking, for 72 hours at 37°C (excitation and emission wavelength 440 nm and 510 nm respectively).

#### AD and PSP tau seed extraction

Tau filaments were extracted from brain tissue from anonymised donors with histopathologically confirmed diagnoses from the Oxford Brain Bank (Ethics approval reference: 15/SC/0639, UK South Central-Oxford C Research Ethics Committee) and Cambridge Brain Bank (under the ethically approved protocol for “Neurodegeneration Research in Dementia” (REC 16/WA/0240)). The donors were an 85-year-old male with confirmed neuropathological diagnosis of PSP and an 81-year-old female with confirmed neuropathological diagnosis of AD. <1.5 g of grey matter was dissected, and the weight recorded. 10X volumes of extraction buffer (800 mM NaCl, 10 mM Tris-HCl, 2.5 mM EDTA, 15% sucrose, 2% sarkosyl, 1% protease inhibitor in 10 ml H_2_O) were added and the tissue was homogenised with a Kinematica Polytron PT2500E Homogenizer. The homogenate was incubated at 37°C for 30 minutes and then transferred to 1.5 ml microcentrifuge tubes. Homogenate was spun for 20 minutes at 18,000 g, at 25°C and then filtered through a 0.45 μM cell strainer. The supernatant was transferred to 1.5 ml ultracentrifuge tubes and spun for 1 hour at 45,000 RPM, at 25°C in TLA55 rotor. The top lipid layer was discarded and the supernatant was transferred to a new tube. The pellets were combined and resuspended in 750 μl of TBS in a new ultracentrifuge tube. The pellets were spun for 1 hour at 45,000 RPM, at 25°C. The supernatant was discarded and 250 μl of TBS per 1 g starting weight was added. The pellet was resuspended by pipetting and sonicating in a water bath.

#### P301S TV tau seeding assay

HEK cells expressing 0N4R P301S tau-venus (TV cells) were plated in 50 μl of OptiMEM per well, at a concentration of 20,000 cells per well, in black 96 well plates (Corning, 3603). P301S tau aggregates were diluted to the appropriate concentration with OptiMEM in a volume of 50 μl per well and 0.5 μl of Lipofectmine 2000 (Fisher Scientific, 11-668-027) was added for each well. The transfection mixture was incubated for 20 minutes in a sterile hood at room temperature. After the incubation, 50 μl of the tau aggregate mix was added to each well. The cells placed in an incubator for 1 hour, and then the Lipofectamine 2000 was neutralised by the addition of 100 μl of complete DMEM. The cells were moved to an IncuCyte S3 Live-Cell Analysis System at 37°C for 72 hours, and pictures were taken every 2-4 hours for analysis.

#### Secondary Seeding assay

30 μl PBS was added with 1X protease and phosphatase inhibitor to each well of a 96 well plate and the cells were mechanically resuspended via pipetting. The cells were transferred to an eppendorf tube where they were freeze-thawed five times. Whole cell lysate was then used as ‘seeding’ material. TV cells were plated as described above, and 1 μl of cell lysate was diluted with OptiMEM in a volume of 50 μl per well and 0.5 μl of Lipofectmine 2000 for each well. The rest of the seeding assay was carried out as detailed above.

#### Transmission Electron Microscopy (TEM)

*In vitro* assembled tau filaments were deposited on glow-discharged 400 mesh form- var/carbon film-coated copper grids (EM Sciences, CF400-Cu) for 40 seconds, and the excess of liquid was drained with Whatman filter paper. The grids were then stained with 2% uranyl acetate for 40 seconds and after draining the excess of liquid, the grids were left to air-dry for at least 30 minutes before image acquisition. Images were acquired at 6,500X, with a defocus value of -1.4 μm with Gatan Orius SC200B or Gatan Ultrascan 1,000 CP CCD detectors using a Tecnai G2 Spirit at 120 kV.

#### P301S TVA tau degradation assay

TVA cells were plated at a concentration of 15,000 cells per well in a volume of 200 μl in black 96 well plates. Lentiviruses carrying tau-RING +/- RING mutations were added and cells were moved to an IncuCyte S3 Live-Cell Analysis System at 37°C for 72 hours. Pictures were taken every 2-4 hours for analysis. Small molecule inhibitors (listed below) were applied at the indicated concentration at 24hrs after application of lentivirus. Alternatively, a DMSO control was applied. The number of aggregates was analysed every two hours for a further 24 hours after the application of inhibitors. The following inhibitors were used: E1 inhibitor TAK-243 (Selleckchem, S8341), VCP inhibitor NMS-873 (Selleckchem, S7285), Proteasome inhibitor MG132 (Sigma, M7449). The DUB inhibitor (Sigma, PR-619) was applied for 6 hrs before removal and fixation of the cells. The starvation assay was carried out by plating cells as above, and adding tau-RING lentivirus for 48hrs. Cells were subsequently starved in HBSS for 5 hours, before being lysed in Western Blot Lysis Buffer (20 mM HEPES, 50 mM potassium acetate, 2 mM EDTA, 200 mM Sorbitol, 1% Triton X-100, 0.1% SDS). 3 wells per condition were pooled before being processed for western blot analysis.

#### WT tau seeding assay

0N3R venus-tau lentivirus was generated as described above. Lentivirus was applied to HEK293T cells, plated in a 6 well plate at 250,000 cells per well, and left to incubate for 2 days to begin expressing the construct. Cells were transferred to a black 96 well plate, and plated at 15,000 cells per well. They were seeded with 1 μl of AD brain derived tau per well as described above in the P301S TV tau seeding assay. The formation of aggregates was tracked using a IncuCyte S3 Live-Cell Analysis System; after 2 days aggregates had formed. The cells were then replated in a second black 96 well plate at 20,000 cells per well in 200 μl complete DMEM, and lentivirus expressing 0N3R tau-RING was applied. The final number of aggregates was counted 72 hrs later. The same assay was carried out to test PSP derived tau aggregates with a 0N4R tau system. In this case PSP derived tau took longer to seed, therefore the aggregates were given 5 days to appear before the application of lentivirus.

#### Primary neuron preparation

Plates were coated with sultrex Poly-L-Lysine and incubated overnight, then washed three times with sterile water and dried. Plating media 100ml (43 ml Neurobasal Plus, 5 ml Horse Serum, 1 ml B-27 plus 50X, 0.5 ml 100X GlutaMAX, 0.5 ml Penicillin/Streptomycin) and maintenance media (50 ml total- 48 ml Neurobasal Plus, 1 ml B-27 plus 50X, 0.5 ml 100X GlutaMAX, 0.5 ml Penicillin/Streptomycin) was prepared, filtered, and refrigerated. Mice were sacrificed, and their brains were dissected and placed in hibernate-A containing plate on ice. Hippocampi and cortices were isolated and washed three times in hibernate-A, followed by a trypsin incubation at 37°C for 20 minutes with intermittent mixing. Post-trypsinization, 1% RNAse was added for a brief incubation, and tissues were washed with warm hibernate-A and plating media. Tissues were triturated, strained through a 70 μm cell strainer, and the single cell suspension was counted with trypan blue using an automated cell counter. Cells were plated at 30,000 cells per well in a 96-well plate with plating media, which was replaced with maintenance media after four hours. Cultures were maintained for up to two weeks in an incubator.

#### Primary neuron tau seeding assay

Primary P301S neurons were infected at day 2 with 1×10^10^ vgs of AAV PHP.eB venus or venus-P2A-tau-RING per well of a 96 well plate. 100 nM tau aggregates were diluted in 25 μl of primary neuron maintenance media and added to each well of a 96 well plate at day 7. Seeding was evaluated at day 14 by fixing the neurons with methanol, and carrying out immunofluorescence staining for AT8 (Fisher Scientific, MN1020) positive aggregates.

#### AAV production

Three confluent 15 cm dishes of HEK293 cells were expanded into ten dishes using complete DMEM and incubated overnight. After changing the media to DMEM + 10% FBS (without Pen-Strep), cells were transfected with 1X AAV plasmid transfection mixture per 15 cm dish (7 μg pUCmini-iCAP-PHP.eB, 7 μg pAAV-CAG-cargo, 20 μg pAdDeltaF6, 1.8ml OptiMEM and 170 μl PEI). 16 hrs post-transfection, media was switched to DMEM + 1% Pen-Strep (no FBS) to inhibit further growth. Cells and media were harvested 48 hrs later; cells were lysed and AAV was precipitated from the supernatant using NaCl and PEG, then pelleted and resuspended in lysis buffer. Additional MgCl_2_ and Benzonase were used for DNA digestion following 3 freeze-thaw cycles. The lysate was centrifuged, and the AAV-containing supernatant was purified using an iodixanol gradient centrifugation. AAV was purified using an iodixanol gradient in a Beckman 70Ti rotor, and spun for 70 minutes at 68,000 RPM at 17°C in a Beckman Ultracentrifuge. The AAV band was extracted from the 40% fraction using a needle and syringe, concentrated using Amicon Ultra tubes with a 100 kDa cut off, and buffer-exchanged to PBS. The final virus concentrate was stored at -80°C. AAV purity and titration were assessed by SDS-PAGE and Coomasie Blue staining, confirming the purity and integrity of capsid proteins VP1, VP2, and VP3.

#### AAV injection of adult mice

For intravenous injection, mice were injected with a volume of 100 μl containing 4×10^11^ vgs of AAV 9P31, or the same volume of PBS alone, in the lateral caudal tail vein. 8 mice were designated per group. Only 6 mice were injected in the VPTR treated group due to insufficient virus. 8 mice were injected with VPTR I18R/M72E, however one mouse developed a severe motor phenotype before the 6-month end point and had to be culled, and so was excluded from analysis. For stereotaxic injection, mice were anaesthetised using 2% isoflurane 1 L/min O_2_. The crown of the head was shaved and cleaned with antiseptic, and the mouse was injected with 0.1 ml Rimadyl s/c. The mouse was placed in the stereotaxic frame and a 1 cm incision was made into the scalp. The skull was cleaned and local anaesthetic mepivicaine was applied to the drill site. After 2 minutes, the drill was zeroed to bregma, and then positioned at the following co-ordinates to inject the frontal cortex: AP: +2.96, ML: +0.75, DV: +0.75 mm. A small 1 mm hole was drilled through the skull, without touching the cortex. The cortex was injected with 5×10^10^ vgs of AAV PHP.eB in a volume of 1.5 μl at a rate of 0.5 μl per minute. After sealing the wound, the mouse was removed from the frame and placed in a recovery chamber. Mice were weighed for a week after the procedure to monitor adverse side effects. No adverse effects were reported from any of the procedures carried out in this study.

#### Sarkosyl insoluble extraction of mouse brain tau

Brains from mice which were intravenously injected were bisected down the midline. One half was taken for fixing, and the other half was used for sarkosyl insoluble extraction and western blot analysis. The cerebellum was removed, and the rest of the hemisphere was weighed (average ~0.2 g). 10X Homogenisation Buffer (8 ml 5M NaCl, 1ml 1M Tris-HCl, 500 μl 0.5M EDTA, 7.5g Sucrose, 1x Halt Protease and Phosphatase inhibitors, 40.5 ml H_2_O) was added w/v. Brains were thoroughly homogenised using an Kinematica Polytron PT2500E Homogenizer and kept on ice. 1 ml was taken for Sarkosyl Insoluble extraction and the rest of the homogenate was frozen at -80°C for further analysis. Sarkosyl was added to 1 ml homogenate in an Eppendorf tube from each brain to make a final concentration of 1%, and left rotating at room temperature for 1 hr. The Eppendorfs were then spun at 18,000 g for 20 minutes at room temperature. The supernatant was pipetted off and transferred to Beckman Coulter 1.5 ml ultracentrifuge tube. The tubes were spun at 45,000 RPM for 1 hour at 20 degrees in a bench top ultracentrifuge (Beckman Optima Max-XP). The supernatant was removed, and the SI pellet was resuspended in 1 ml of 1X TBS, and spun again at 45,000 RPM for 1 hour at 20 degrees. The supernatant was removed, and the pellet was resuspended in 50 μl of 1X TBS with 1X Halt protease and phosphatase inhibitors, and stored at -80°C. To run on a western blot, SI samples were diluted 1:4 in TBS. Homogenate was diluted 1:10 in TBS.

#### Cryo-sectioning adult mouse brains

Brains from stereotaxically injected mice were taken whole, while brains from intravenously injected mice were bisected. All brains were fixed in 4% PFA at 4°C for 24 hours, then submerged in 30% sucrose in PBS for 3 days. Brains were embedded OCT (Sakura, 94-4583) within a cryo-sectioning mould and cooled on dry ice. Once solidified, the moulds were stored at -80°C for 2 hrs before use. Brains were sectioned at 30 μm thickness, and stored in cryoprotectant (25% glycerol, 30% ethylene glycol, 45% PBS) at -20°C. Before staining, sections were transferred to PBS and washed 3x to remove cryoprotectant.

#### Immunohistochemistry

Adult brain slices, three per brain and spaced approximately 150 μm apart, were placed in 24-well plates containing 400 μl of blocking buffer (PBS, 3% Goat Serum, 0.5% Triton X-100) and incubated for 1 hr at room temperature with shaking. After removing the blocking buffer, slices were incubated overnight at 4°C with 400 μl of primary antibody mix (blocking buffer + primary antibodies), followed by washing three times in PBS. The secondary antibody mix (blocking buffer + secondary antibodies + DAPI) was pre-cleared by centrifugation at 20,000 RPM for 10 minutes and then added to the slices for 1 hr at room temperature in the dark with gentle shaking, followed by three PBS washes. Slices were mounted on glass slides using a PBS bath, covered with Prolong Diamond Antifade Mountant, and topped with a coverslip. Slides were dried overnight in the dark and were imaged using an Olympus Slide Scanner. Primary neurons, post methanol fixation, followed a similar staining protocol and were imaged in PBS using an Incucyte. For confocal imaging of the HEK cell assays, HEK cells were plated in μ-Slide 8 Well Chamber Slides (iBidi GmbH). The assays were performed as described above, and cells were fixed with 4% paraformaldehyde in PBS for 15 minutes, before being washed with PBS, and incubated with 0.1% Triton X-100 + 5% goat serum in PBS for 1 hr. The same protocol was subsequently followed as above. Slides were imaged using a confocal setup (Zeiss 880 Airyscan). Quantification analysis was performed using ImageJ software.

#### Live cell imaging of BHK cells

Live cell imaging was performed using μ-Slide 8 Well Chamber Slide-wells (iBidi GmbH) in the Nikon Ti2 microscope. BHK-tau-venus/tau-mcherry-ring cells were plated at 80% confluence the day before and seeded the day after with in-vitro aggregated tau as above. Real time imaging was carried out at 37 °C in a 5% CO2 humidified chamber using Nikon Ti2 microscope with 40x objective. Image reconstruction was carried out using NIS-Elements software, while quantification analysis was performed using ImageJ.

#### Western blot

HEK cells and primary neurons were lysed in Western Blot Lysis Buffer (20 mM HEPES, 50 mM potassium acetate, 2 mM EDTA, 200 mM Sorbitol, 1% Triton X-100, 0.1% SDS) containing 1% Halt protease and phosphatase inhibitors, incubated on ice for 20 minutes, then centrifuged at 14,000 g. The supernatant was used directly or frozen. Mouse brain homogenates were diluted 1:10 in TBS without centrifugation. For Western blotting, protein samples mixed with NuPAGE LDS sample buffer were heated at 95°C for 3 mins, run on a NuPAGE Bis-Tris gel, and transferred to a PVDF membrane using a Trans-Blot Turbo system. Membranes were blocked in 5% milk-TBST, incubated with primary antibodies (1:1000 in 5% milk-TBST) overnight at 4°C, washed, then incubated with secondary antibodies-HRP (1:5000 in 5% milk-TBST). Detection was performed using either fluorescent or HRP conjugated antibodies and imaged accordingly.

#### Proteomic analysis

For mass spectrometry analysis, 100 μg of each brain lysate was diluted to 1 μg/μL using 25 mM AMBIC. Cysteines were reduced by adding DTT to a final concentration of 4 mM and heating the samples to 60°C for 10 mins. To prevent cysteine re-oxidation, iodoacetamide was added as an alkylating reagent to a final concentration of 14 mM followed by incubation at room temperature in the dark for 45 minutes. Digestion was carried out semi-automatically on a Kingfisher Apex using the Protein Aggregation Capture method adapted from Batth et al.^49^ Reduced and alkylated samples were transferred to a 96 well plate and precipitated by adding acetonitrile to a final concentration of 70% (v/v). Washed MagResyn Hydroxyl microparticles from Resyn Biosciences were immediately added to the samples at a ratio of 1:4 (protein:bead) to promote protein precipitation and on-bead aggregation. Beads with the aggregated proteins were washed 3X with 100% acetonitrile, followed by 2X washes with 70% ethanol. In-bead digestion was performed on the Kingfisher Apex by adding 1 μg of trypsin to 100 μL of 25 mM AMBIC containing 0.2% RapiGest detergent (Waters) and incubating for 1 hr at 47°C. Overnight digestion was then carried out at 37°C on an Eppendorf ThermoMixer C for an additional 16 hr. Magnetic beads were removed and peptides were acidified with the addition of trifluoroacetic acid to a final concentration of 0.5% (v/v). The acidified tryptic digest was then centrifuged at 13,000 x *g* for 15 mins to remove RapiGest degradation by-products and the supernatant subjected to LC-MS/MS analysis.

LC-MS/MS was performed on an Vanquish Neo UHPLC (ThermoFisher Scientific, San Jose, USA) hyphenated to an Orbitrap Eclipse mass spectrometer (ThermoFisher Scientific, San Jose, USA). Peptides were trapped on a C18 Acclaim PepMap 100 (5 μm, 300 μm x 5mm) trap column (ThermoFisher Scientific, San Jose, USA) and separated on a C18 Aurora Ultimate TS (25cm x 75 μm) column (IonOpticks, Australia) over a gradient of solvent B (A: 0.1% (v/v) formic acid; B: 80 % (v/v) acetonitrile, 0.1% formic acid) from 3% to 25% B over 135 mins followed by a 25% to 45 % B over 45 mins. MS1 full scans were acquired in the Orbitrap mass analyser at a resolution of 120,000 (AGC target of 4e5 ions with a maximum injection time of 50ms) and followed by MS2 in a data-independent acquisition setting composed of 41 staggered variable width windows covering 400-900 m/z. MS2 DIA scans were acquired in the Orbitrap mass analyser at a resolution of 30,000 with a maximum injection time of 54ms and an HCD collision energy of 30%.

Raw data were imported and processed in Spectronaut 18.0 (Biognosys) using stringent criteria as set out by Baker et al.^50^ Raw files were searched against ***Mus musculus*** protein sequences downloaded from UniProt in July 2023 (UP000000589_2023_07_04) with the human tau sequence appended to the mouse database. Differential abundance testing was performed using unpaired t-test with group-wise testing correction. All raw files and data analysis files produced in Spectronaut have been uploaded to PRIDE (https://doi.org/10.1093/nar/gkab1038) under the accession number PXD052897.

MouseWalker phenotypic testing A MouseWalker was constructed by the LMB mechanical workshop, from schematics presented by Mendes et al.^32^ Mice were habituated to the MouseWalker and trained by attaching the home cage to the distal end of the MouseWalker, allowing the mice to walk towards it as an incentive. Mice were allowed to rest in the home cage for 1 minute before repeating the procedure 5X. For each experimental time point, an average of 3 videos of the mouse traversing the walkway were collected. Up to 10 attempts were allowed per mouse to obtain a video of the mouse walking across the length of the walkway uninterrupted. Mice were tested every 2 weeks from 4-6 months. For VPTR I18R/M72E and PBS treated mice, one mouse was excluded from analysis in each group due to the inability to obtain a video of the mouse walking at the 6-month time point. Videos were analysed by training a neural net from open-source software provided by DeepLabCut.^33^

## Quantification and Statistical Analysis

### Image analysis

Live-cell images were automatically processed using the IncuCyte S3 Live-Cell Analysis System, which quantified both the total cell area and number of tau puncta per well. The cell area was determined by phase contrast imaging, whilst tau aggregates were detected by segmentation analysis using the fluorescent signal from tau-venus positive puncta. The number of puncta was divided by the cell area to allow for variation in confluence between wells. To allow direct comparison between conditions, and to combine the data from multiple biological replicates, all values were normalised to the mean of the control condition for each experiment. Graphs presenting aggregate levels as ‘Relative level of tau aggregates (%)’, are therefore presenting data analysed as ((tau puncta/cell area)/control mean) x 100. The tau puncta in Figures 1E, 1F, 2, and 3F were analysed by the IncuCyte segmentation software. Due to the smaller size of the aggregates, the tau puncta in Figures 1C and 3D were analysed using the comdet plugin in Fiji,^48^ which is a specialised plugin for finding and detecting small, bright intensity spots. The primary neuron seeding assay was analysed for tau aggregation by creating a binary mask to segment the AT8 positive area per image, as the presence of small and large densities of aggregates precluded the use of comdet for overall quantification. AT8 positive area was divided by the NeuN count (neuronal nuclei) per image, and was normalised to the control mean for each experiment to correct for the variation between biological replicates (Figure 4B). ‘Relative AT8 positive area (%)’ therefore represents the ((AT8 positive area/NeuN count)/control mean) x 100. AT8 positive neuronal cell bodies were counted manually per image, normalised to NeuN count, and plotted as (AT8 Positive cell bodies/NeuN count) x 100 (Figure S3H). AT8 puncta (present predominantly in the neuronal processes) were segmented using the comdet plugin in Fiji, and expressed as ‘Relative AT8 positive puncta (%)’ by calculating ((AT8 positive puncta/NeuN count)/control mean) x 100 (Figure S3I). The AT8 positive area in mouse brain sections (Figure 5C) was analysed using a binary threshold and % positive area was calculated for each ROI: (AT8 positive area/total area) x 100 (Figure 5C).

### DeepLabCut Analysis

For body part tracking, a total of 1050 MouseWalker videos were analysed using the open-source software package by DeepLabCut.^33^ A residual neural network with a ResNet-50 architecture^51,52^ with default parameters was trained using videos randomly sampled from all treatment groups. Every 20^th^ frame within each training video was extracted and mouse footprints manually labelled as either front-left, front-right, back-left or back-right if in contact with the walkway. Training was performed on a custom-built workstation with an Intel Xeon Gold 5220 CPU, 128 GB RAM and four NVIDIA RTX A6000 48GB GPUs with NVLinks; with a batch size of 8. We found that our test error was 4.26 pixels and then used a p-cut off of 0.6 to condition XY coordinates for future analysis. The trained neural network was then used to analyse all videos acquired.

### Statistics

All statistics were performed using the Prism 10 software unless otherwise indicated. ‘N’ indicates biological replicates, ‘n’ indicates technical replicates. All graphs plotted with the mean and standard deviation unless otherwise indicated. Statistical test use determined by distribution and group size, denoted in the figure legends.

## Supplementary Material

Supplemental figures

## Data Availability

Mass spectrometry datasets have been deposited in the Proteomics Identification Database (PRIDE) and are publicly available as of the date of publication. Accession numbers listed in the key resources table. This paper does not report original code. Any additional information required to reanalyze the data reported in this paper is available from the lead contact upon request.

## In brief

RING-Bait technology hijacks the process of templated aggregation to recruit ubiquitination machinery and selectively degrade pathogenic tau assemblies without generating a specific binder, thereby reducing tau pathology and improving motor function *in vivo*.

## Highlights

- RING-Bait technology turns aggregate sequences into potent aggregate degraders
- RING-Bait degraders remove pre-existing aggregates and prevent new ones from forming
- RING-Bait degraders prevent seeded aggregation from human AD and PSP tau aggregates
- RING-Bait degraders reduce tau pathology and improve motor function *in vivo*
