# Supplementary material for: Co-opting templated aggregation to degrade pathogenic tau assemblies and improve motor function
Source: Cell. Author manuscript; Available in PMC 2024 Nov 19. (PMC7616835; doi:10.1016/j.cell.2024.08.024)
Supplement: Supplemental figures [file EMS200070-supplement-Supplemental_figures.pdf]

Supplemental figures

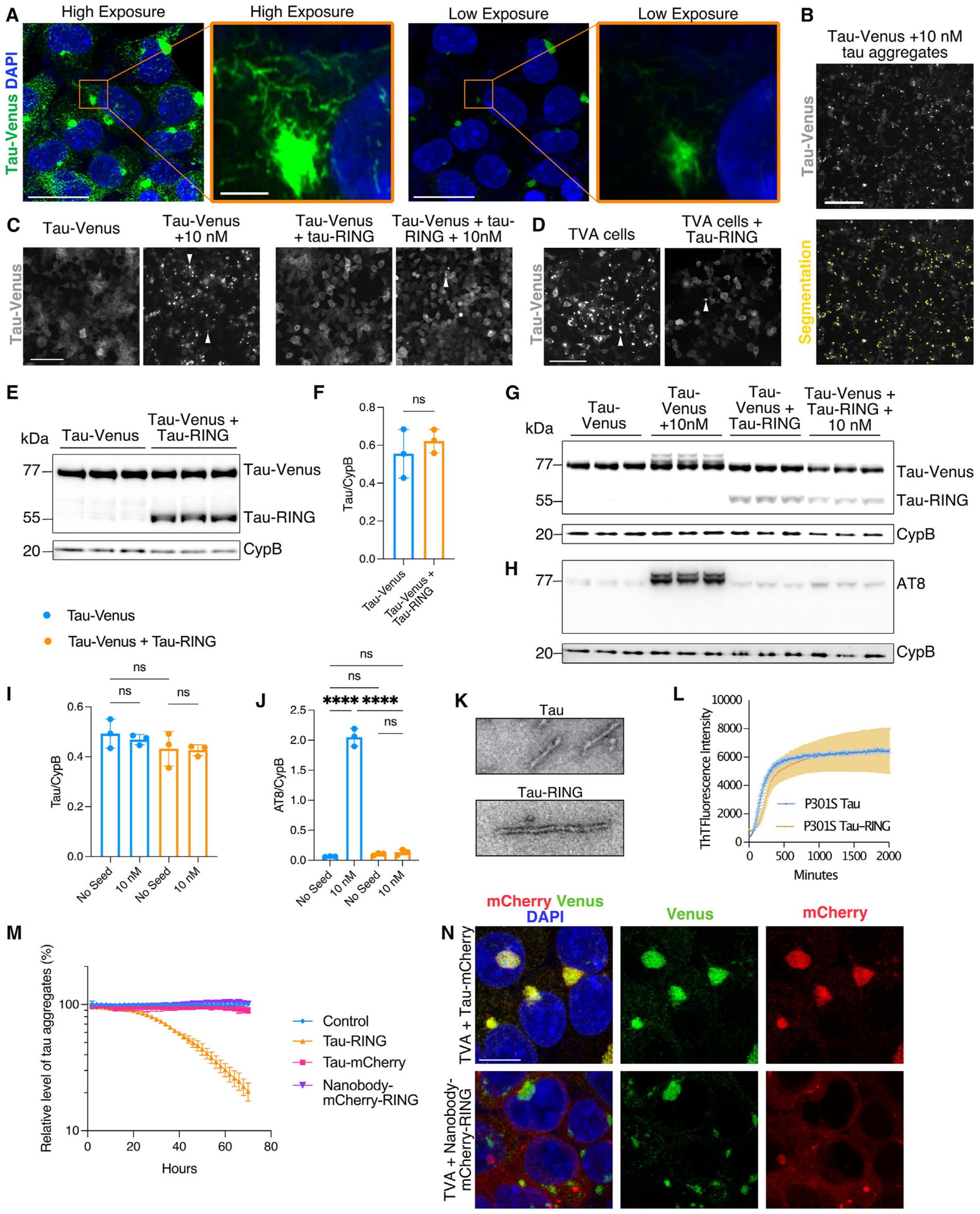

(legend on next page)

Figure S1. Live-cell, biochemical, and immunohistochemical analysis of HEK293 tauopathy model systems, related to Figure 1

(A) High and low exposure versions of the "tau-venus + 10 nM" condition in Figure 1B. Enlarged regions allow visualization of different densities of fibrillar tau aggregates. Scale bars, 25 and 3 μm.

(B) Representative live-cell image of HEK293 cells expressing P301S tau-venus, seeded with 10 nM tau aggregates. Bright puncta form, which can be segmented and quantified (yellow dots are a representative segmentation). Scale bar, 500 μm.

(C) Representative live-cell images for conditions presented and quantified in Figure 1B and 1C. Scale bars, 250 μm.

(D) Representative live-cell images for conditions presented and quantified in Figure 1D and 1F. Scale bars, 250 μm.

(E) Western blot of HEK293 reporter cells expressing tau-venus ± tau-RING, probed for tau, and loading control CypB.

(F) Quantification of (E).

(G) Western blot of cells expressing tau-venus ± tau-RING ± 10 nM tau aggregates delivered with Lipofectamine 2000. Blot probed for tau and loading control CypB.

(H) Western blot of cells treated as in (G), probed for hyperphosphorylated tau (AT8), and loading control CypB.

(I) Quantification of tau-venus in blot (G).

(J) Quantification of AT8 in blot (H).

(K) TEM of tau and tau-RING fibrils aggregated with heparin.

(L) Tau and tau-RING protein aggregation kinetics in the presence of heparin, visualized with thioflavin T (ThT).

(M) TVA cells treated with lentivirus expressing tau-RING, tau-mCherry, or a nanobody-mCherry-RING construct. Nanobody binds yeast GCN4 and therefore acts as a non-specific control for an active RING construct. $N = 3$.

(N) representative confocal images of TVA cells treated with tau-mCherry or the nanobody-mCherry-RING construct. Tau-mCherry co-localizes with the tau-venus aggregates, whereas the nanobody-mCherry-RING construct does not. Statistical significance for (F) determined by an unpaired t test. Statistical significance for (I) and (J) determined by one-way ANOVA and Tukey's multiple comparisons test. ****$p < 0.0001$. ns, non-significant.

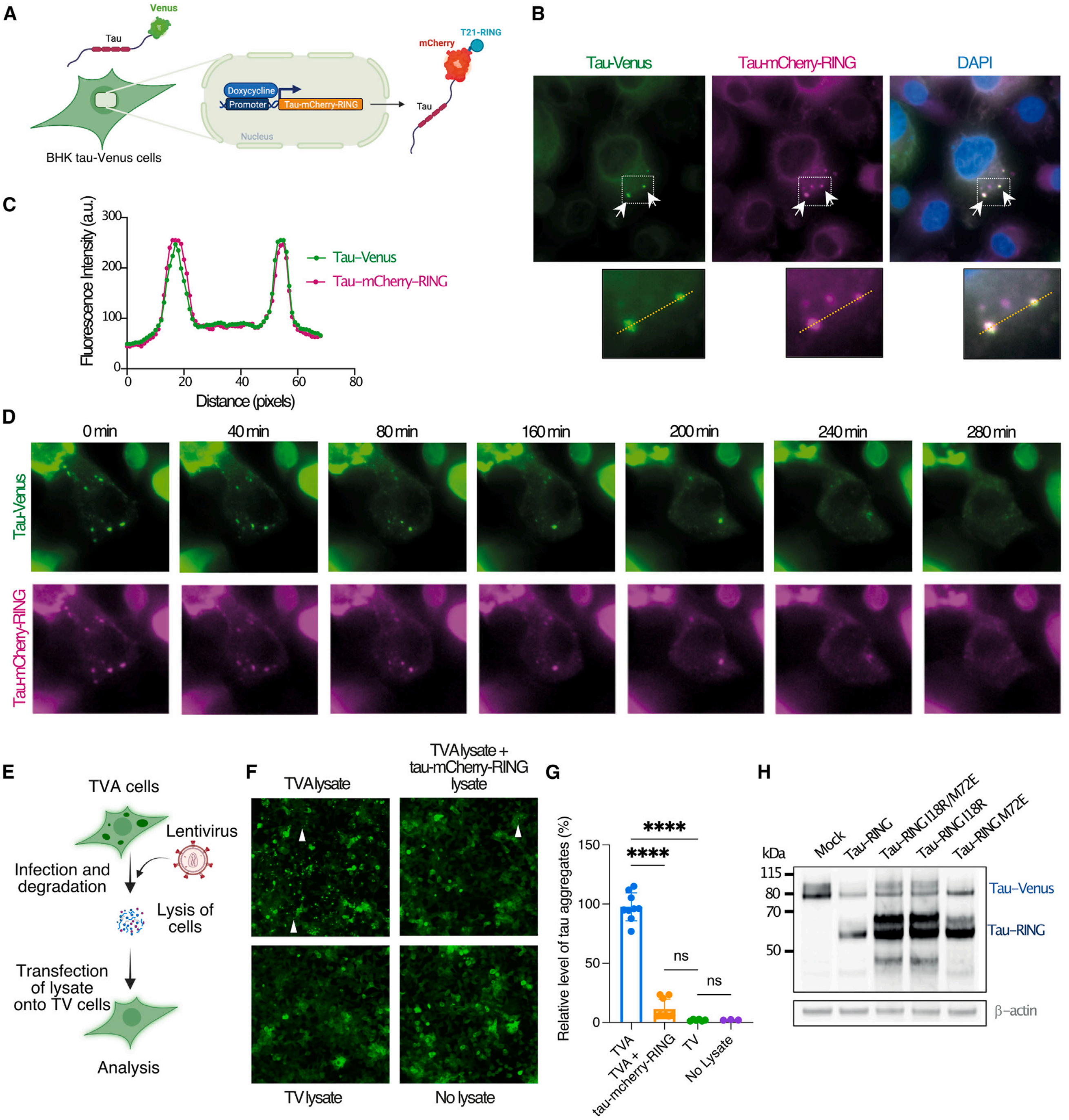

Figure S2. Tau-RING co-localizes with aggregates prior to removal and reduces seed-competent species, related to Figure 1

(A) BHK cells expressing P301S tau-venus were infected with doxycycline-inducible tau-mCherry-RING.

(B) Upon induction of tau-mCherry-RING expression and seeding of cells with pre-formed P301S tau aggregates, tau-venus and tau-mCherry-RING were observed to co-localize in aggregates.

(C) Quantification of co-localization of tau-venus and tau-mCherry-RING.

(D) Time course of tau-mCherry-RING causing the removal of tau-venus positive aggregates in a BHK cell.

(E) Schematic of the HEK293 secondary seeding assay. TVA cells were infected with a lentivirus carrying tau-mCherry-RING. After degradation of the aggregates occurred, cells were lysed, and homogenate was transfected into fresh TV reporter cells using Lipofectamine 2000. Lysate was also taken from uninfected TVA cells and TV cells. Seeding in the TV cells was analyzed 72 h later.

(legend continued on next page)

(F) Representative images of secondary seeding in TV cells upon transfection with lysate from TVA, TVA + tau-mCherry-RING, and TV cell lysate. White arrows denote aggregates.

(G) Quantification of secondary seeding in TV cells upon transfection with lysate from TVA, TVA + tau-mCherry-RING, and TV cell lysate. $N = 3$.

(H) TVA cells treated with lentivirus encoding for tau-RING ± I18R, M72E, or I18R/M72E mutations probed for tau protein 72 h after infection. Statistical significance for (G) determined by one-way ANOVA and Tukey's multiple comparisons test. ****$p < 0.0001$. ns, non-significant.

CellPress
OPEN ACCESS

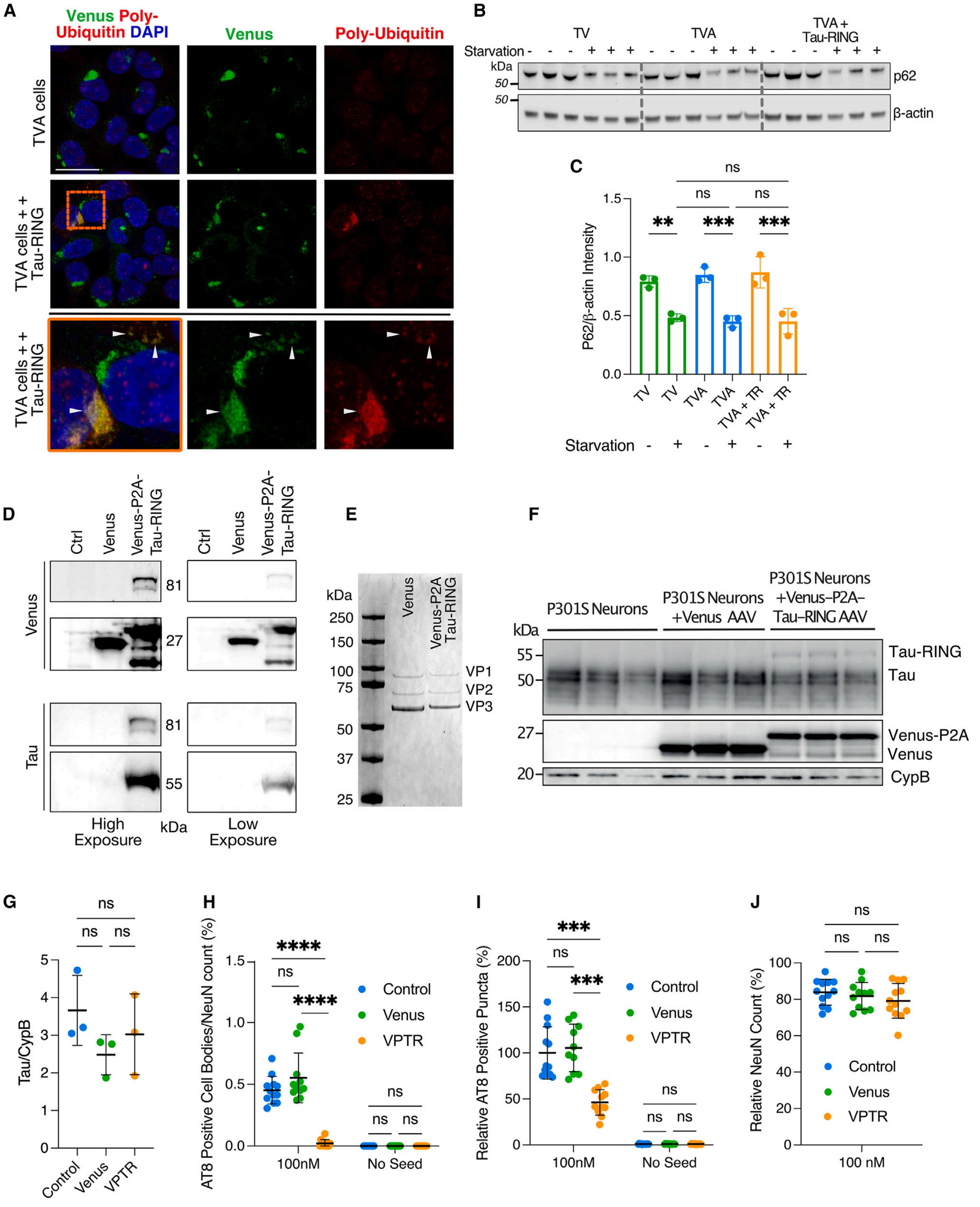

(legend on next page)

Figure S3. Tau-RING induces poly-ubiquitination of tau-venus aggregates without affecting autophagy and is effective in primary neurons, related to Figures 2 and 4

(A) Representative images of TVA cells treated with a lentivirus that expresses tau-RING in the presence of the DUB inhibitor PR-619, stained for poly-ubiquitin chains (FK2 antibody). The enlarged section highlights areas where poly-ubiquitin chains and tau-venus positive aggregates co-localize. Scale bar, 25 μm.

(B) Western blot of TV cells, TVA cells, and TVA cells + tau-RING lentivirus. Cells were cultured in full media or starved for 5 h before blotting for p62 to observe the induction of autophagy. *N* = 3.

(C) Quantification of cells treated as in (B). No significant difference in p62 levels was observed after starvation between cells that did not have aggregates (TV cells), cells with aggregates (TVA cells), and TVA cells treated with tau-RING for 48 h.

(D) Western blot showing HEK293 cells transfected with the pAAV genome plasmid containing venus-P2A-tau-RING, venus, or the empty vector (Ctrl). The blot was probed for venus protein and tau protein. High and low exposures of blots presented.

(E) Coomassie staining of AAV purified using an iodixanol gradient. Capsid proteins VP1, 2, and 3 can be visualized without detectable contaminants.

(F) Western blot of primary neuron cultures infected with venus AAV PHP.eB or venus-P2A-tau-RING AAV 1×10^{10} vgs per well, or untreated, at DIV2 and processed at DIV14. Samples were probed for tau, venus, and loading control CypB.

(G) Quantification of total tau levels in (F).

(H) AT8-positive cell bodies in primary neuron cultures from Figure 4B. *N* = 3.

(I) Quantification of AT8-positive tau puncta found in neuronal processes from primary neuron cultures treated as in Figure 4B. *N* = 3.

(J) NeuN count from primary neurons treated as in Figure 4B, normalized to the NeuN count for each condition without tau aggregates. *N* = 3. Statistical significance for (C), (G), and (J) determined by one-way ANOVA and Tukey's multiple comparisons test. Statistical significance for (H) and (I) determined by two-way ANOVA and Sidak's multiple comparisons test.****$p < 0.0001$. ns, non-significant.

CellPress
OPEN ACCESS

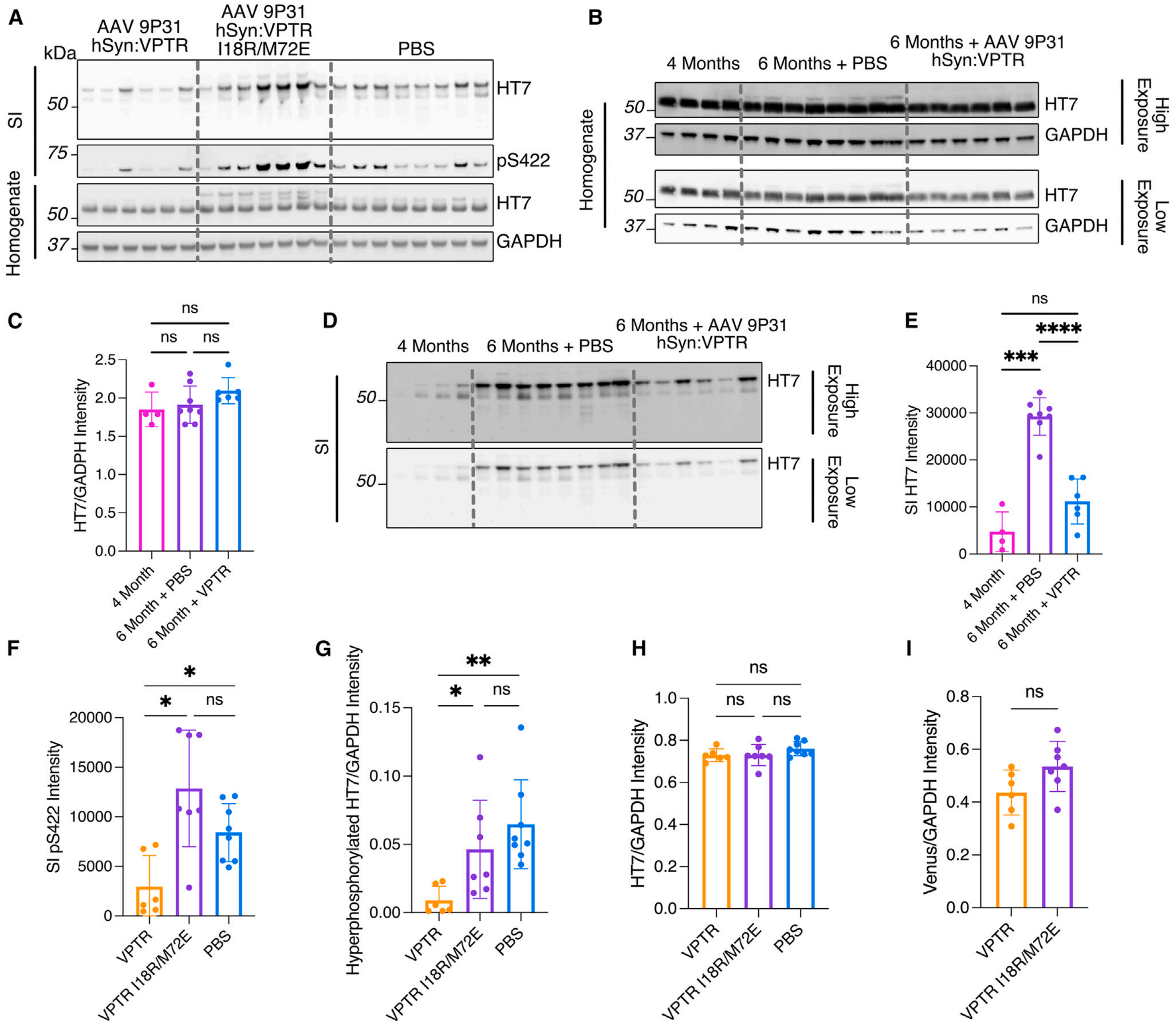

Figure S4. Analysis of 4- and 6-month-old P301S tau transgenic mice, related to Figure 5

(A) Low exposure blots from Figure 5D.

(B) Whole brain homogenate tau levels from 4-month-old mice were compared with 6-month-old mice treated with VPTR AAV 9P31 or PBS. High and low exposure presented to enable visualization of the higher molecular weight band in the 6-month-old PBS-treated mice, which is absent in 4-month-old mice.

(C) Total tau levels from the HT7 staining were quantified from (B) and showed no significant difference.

(D) SI extraction of tau from 4-month-old P301S mice compared with 6-month-old mice treated with VPTR AAV 9P31 or PBS.

(E) SI tau levels from the HT7 staining in (D) were quantified and showed a significant increase in SI tau from 4 to 6 months.

(F) Quantification of hyperphosphorylated tau positive for pS422 from Figure 5D.

(G) Quantification of hyperphosphorylated HT7 band in homogenate western blot from Figure 5D.

(H) Quantification of main HT7 band in homogenate western blot from Figure 5D.

(I) Quantification of venus protein from western blot in Figure 5D. Statistical significance for (C) and (E) determined by one-way ANOVA. Statistical significance for (F)–(H) determined by Brown-Forsythe and Welch ANOVA. Statistical significance for (I) determined by unpaired t test. *$p < 0.05$, **$p < 0.01$, ***$p < 0.001$, ****$p < 0.0001$. ns, non-significant.

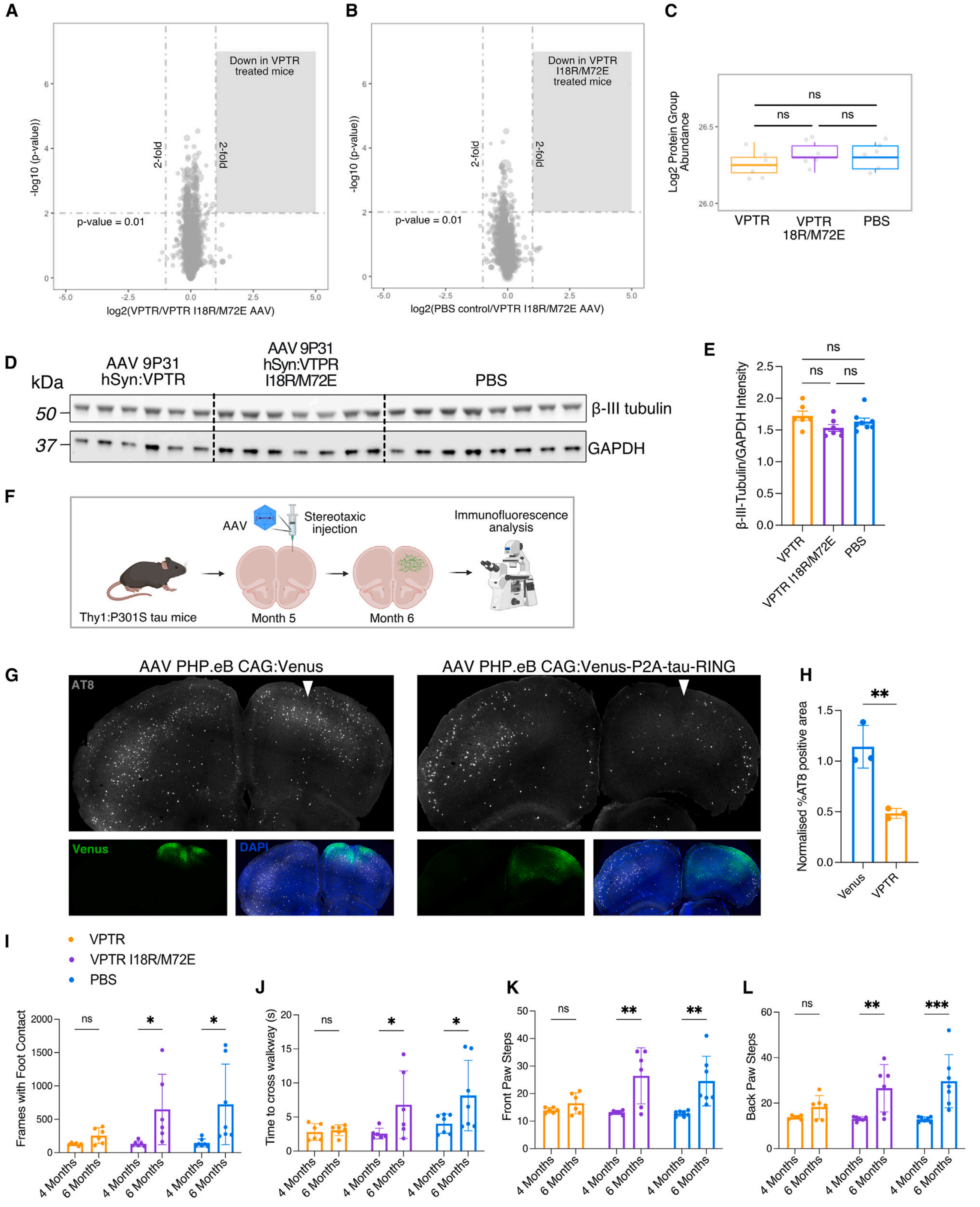

(legend on next page)

Figure S5. Biochemical and behavioral analysis of tau-RING-treated mice, related to Figure 5

(A and B) Mass spectrometry data comparing protein abundance from VPTR- and VPTR I18R/M72E-treated mice and from PBS control and VPTR I18R/M72E-treated mice. Differential abundance testing was performed using an unpaired t test with group-wise testing correction. Proteins were rejected if abundance values were present in less than 3 samples per group. *N* = 6 mice from each condition. Each point of the volcano plot corresponds to a different protein entry scaled to the number of unique peptides identified.

(C) Abundance of β-III-tubulin in VPTR-treated mice from mass spectrometry data.

(D) Western blot of mice treated as in Figure 5, probed for β-III-tubulin and loading control GAPDH.

(E) Quantification of western blot in (D).

(F) Schematic of stereotaxic injection of P301S mice at 5 months, with AAV PHP.eB CAG:venus or venus-P2A-tau-RING (VPTR). Mice were injected at 5 months and culled at 6 months to analyze tau pathology.

(G) Representative images of mice treated as in (F). White arrows denote injection site.

(H) AT8-positive area of the injected hemisphere, normalized to the contralateral, uninjected hemisphere. *N* = 3 mice per condition.

(I) Analysis of the frames per video in which feet were in contact with the MouseWalker platform at 4 months (before treatment) and at 6 months (after treatment).

(J) Time for mice to cross the MouseWalker platform at 4 and 6 months.

(K) Number of front paw steps to cross the MouseWalker platform at 4 and 6 months.

(L) Number of back paw steps to cross the MouseWalker platform at 4 and 6 months. Statistical significance for (E) determined by Brown-Forsythe and Welch ANOVA, and (H) determined by unpaired t test. Statistical significance for (I)–(L), determined by two-way ANOVA and Sidak's multiple comparisons test. $^{*}p < 0.05$, $^{**}p < 0.01$, $^{***}p < 0.001$, $^{****}p < 0.0001$. ns, non-significant.
